# Victim impact statements limit the effects of perspective taking on juror decisions

**DOI:** 10.3389/fcogn.2024.1430999

**Published:** 2024-11-20

**Authors:** Jeanine Lee McHugh Skorinko, Sean Laurent, Emily Bendremer, Kaitlyn Schneider, Valicia Hale, Alisionna Iannacchione, Melissa Paris, Gizem Demircioglu, Kyla Wesley, Julia LaValley, Angelica DeFreitas, Jeremy Blumenthal

**Affiliations:** ^1^Psychological and Cognitive Sciences Program, Worcester Polytechnic Institute, Worcester, MA, United States; ^2^Department of Psychology, The Pennsylvania State University (PSU), University Park, PA, United States; ^3^College of Law, Syracuse University, Syracuse, NY, United States

**Keywords:** victim impact statements, perspective taking, mock jurors, decision making, death penalty

## Abstract

Past work suggests that victim impact statements (VISs) encourage jurors to take victims' perspectives, but this has not been explored empirically. Across four experiments (total *N* = 881), the present research examines the effects of perspective taking and the impact of the crime expressed in VISs on juror perceptions of defendants. In Experiment 1, mock jurors read a capital murder case that prompted them to perspective take (or not) and included VISs that were high or low in impact of the crime on the victims. Results indicate that the impact of the crime expressed in the VISs influenced perceptions of culpability for the defendant, but perspective taking did not. Experiment 2 used an armed robbery case, and the results again showed that the higher impact of the VIS led to seeing the defendant as more culpable, but perspective taking did not. Experiment 3 examined whether the type of perspective taking mattered (imagining self vs. defendant) when VISs were also presented. Those using the self during perspective taking found the defendant less culpable compared to non-perspective takers. Experiment 4 examined whether priming perspective taking influenced decisions. While high-impact VISs resulted in more death penalty sentences than low-impact VISs, priming perspective taking did not. Overall, the impact of the crime expressed in VIS typically influenced the perceptions of the defendants. In contrast, perspective taking had limited effects. These findings contribute to our understanding of VISs in the courtroom and may be useful for attorneys when presenting cases and advising clients.

## Introduction

Victim impact statements (VISs) describe how crimes have impacted someone's life. The widespread use of VISs in the courtroom highlights the need to better understand the effect that impact (including emotional impact) has on juror decision-making (Bandes and Blumenthal, 2012; Blumenthal, 2009, 2005). Although some research has found that the impact of the crime on the victim moderates the effect of VISs on death penalty decisions (Myers and Arbuthnot, 1999; Mitchell et al., 2016; Myers et al., 2002; Salerno, 2021), other research has not. A recent meta-analysis concluded that more research is needed to better understand the effects of VISs on legal decision-making, including mediators and moderators (Kunst et al., 2021).

One important but unexamined factor to consider in relation to VISs is perspective taking, particularly because the target of perspective taking matters. That is, prior work has found that perspective taking with a defendant *decreased* perceptions of guilt, culpability, and recidivism, but taking victims' perspectives *increased* perceptions of defendants' guilt, culpability, and recidivism (Skorinko et al., 2014). This implies that perspective taking with defendants might mitigate VIS effects, but perspective taking with victims might enhance them.

However, equally plausibly, taking the defendant's perspective may fail to impact dependent measures (or might lead to a reversed effect when a VIS is presented), as research has also shown that perspective taking can backfire (Epley et al., 2004; Frantz and Janoff-Bulman, 2000; Hodges et al., 2018; Laurent and Myers, 2011; Skorinko and Sinclair, 2013). For instance, perspective takers can anchor onto available information, which can lead to counterintuitive effects (Skorinko and Sinclair, 2013), and perspective taking may increase prejudicial attitudes due to increasing self-other overlap with the target of perspective taking (Laurent and Myers, 2011). The process of perspective taking can also, at times, highlight differences in how individuals see the world (Hodges et al., 2018), which could limit the extent to which jurors feel capable of taking the perspective of a defendant when a VIS is presented. Thus, the current research examines the effects of perspective-taking targets and VISs on judgments of guilt/sentencing, culpability, and recidivism.

### VISs in the courts

Debates surround the use of VISs in court. In the Supreme Court case *Booth v. Maryland*,^1^ Booth was convicted of murdering an elderly couple. During the sentencing phase, the couple's family gave VISs in accordance with a Maryland state statute requirement for felony cases (Md.Ann.Code, Art. 41, § 4-609(c), 1986). Booth was given the death penalty. On appeal, Booth argued that the VISs elicited a prejudicial emotional response, but courtroom decisions should be based on reason (482 U.S. at 504). The court held that jurors considering VISs during sentencing were biased by information about the defendant's character, violating the Eighth Amendment. This outcome was reaffirmed in *South Carolina v. Gathers*,^2^ where Gathers argued that the prosecution painted the victim in an overly positive way that was irrelevant and prejudiced the jury. The court's ruling extended the ruling in *Booth* to include statements about the victim made by the prosecutor (Myers et al., 2006).

This ruling was reversed in *Payne v. Tennessee*.^3^ In this case, Payne was convicted of murdering a mother and daughter, and the grandmother gave a VIS on the impact the deaths had on the family. The court reasoned that because defendants are permitted to present evidence that speaks to their character to argue that they do not deserve the death penalty, victims should also be allowed to share their experiences (501 U.S. at 817). This set the stage for the Crime Victims' Rights Act (2004), part of which gave victims the right to share how they were impacted by a crime at public court proceedings; see 18 U.S.C.S. § 3771(a)(4).

### Influence of VIS effects on jurors

Although VISs are allowed, their effects are debated (Myers and Greene, 2004). Some argue that VISs are important because they allow victims to be involved in court proceedings, may provide healing for victims, and provide insight into the impacts of a crime (Cassell, 2009; Erez, 1994; Roberts and Erez, 2004). Yet VISs are also criticized because they dehumanize defendants (Bandes, 1996), limit defendants' rights to due process (Schneider, 1992), and may negatively influence sentencing (Hill, 2005).

A recent meta-analysis investigating VISs included 36 studies (31 experiments; five case file studies) and concluded that more research is needed because there was not enough evidence to draw conclusions about the systematic impacts of VISs in the legal system (see Kunst et al., 2021). In this meta-analysis, most experimental research examined death penalty decisions (24 experiments), with fewer experiments exploring sentencing (seven experiments) or guilt decisions (three experiments). Of the seven experiments exploring sentencing, five (71%) found that VISs lead to harsher sentences, and two (29%) experiments found no effects of VISs on sentencing. However, for experiments investigating the death penalty, 7 (29%) found that the death penalty was assigned more when VISs were presented, but 17 experiments (71%) showed no effect for VISs on death penalty recommendations. While some research shows that these findings may be due to participants' attitudes toward the death penalty (Butler, 2008; Luginbuhl and Burkhead, 1995), other research (conducted with undergraduate students, law students, and community members) did not find that a death penalty qualification explained the results (Blumenthal, 2009; Myers and Greene, 2004). In the five criminal case studies reported, there were no associations between VISs and sentencing or death penalty decisions (the cases were in the United States or Australia; Kunst et al., 2021). One potential explanation is that judges in the United States rely on federal sentencing guidelines (Schuster and Propen, 2010), so effects may be difficult to detect. Likewise, study type may play a role. Although field and case studies have more real-world applicability, they cannot match the high control of experimental designs (Salerno and Bottoms, 2009). Still, a recent study examining more than 1,000 sentencing decisions in actual cases found an increased likelihood of VISs being presented in cases in which the crime was more severe and that the way VISs were presented (orally vs. written) and the number of statements presented were associated with increased sentencing lengths (Dufour et al., 2023). The meta-analysis also found no consistent mediators or moderators for the effects of VISs on legal decisions (Kunst et al., 2021). Although numerous mediators and moderators have been examined (e.g., negative affect, need for affect, gender identities, gruesome photos, impact and/or emotional harm to the victim, and emotion expressed in the VIS), few experiments have paired the same mediators or moderators with the same outcome variable (e.g., guilt, sentencing, death penalty), making it difficult to draw strong conclusions.

### Impact of crime on the victim in VISs

One reason VISs are highly debated is because they convey the impact the crime has had on the victim. This impact is broadly defined as the severity of the crime, the emotions or affect disclosed in VISs, or the explanation of harm to the victim (i.e., emotional, psychological, physical, and financial). It is theorized that the impact expressed in VISs will influence juror decision-making (Myers et al., 2013). When looking at whether the crime had mild or severe impacts on the victim (or the victim's family), the severity of the impact moderated the effect of VISs on death penalty decisions (Mitchell et al., 2016; Myers et al., 2002), such that more severe impacts increased death penalty recommendations. But this effect may depend on attitudes toward the death penalty, as harm severity only influenced death penalty decisions when jurors had positive attitudes toward the death penalty in one study. When the impact expressed in the VIS focused on harm to the victim, one study found that jurors who saw VISs with severe emotional harm gave harsher sentences than those who saw VISs with mild emotional harm—but there was no difference between severe emotional harm and VISs with no emotional harm (Nadler and Rose, 2003). Other studies found that VISs that expressed any harm, whether emotional or physical, resulted in harsher sentences than when no VIS was presented (Wevodau et al., 2014a). Likewise, mock jurors are sensitive to whether the emotional content in VISs matches the severity of a crime (i.e., victims who present emotional VISs in less severe crimes are perceived as less credible; Lens et al., 2016).

Research also shows that jurors feel moral emotions when they believe someone has been treated immorally (Laurent et al., 2016; Salerno, 2021). These moral emotions can be negative and condemning in nature (e.g., anger) associated with the desire to punish or blame or can be positive and involve concern for others (e.g., empathy). Regarding the discrete emotion of anger, jurors who saw anger in VISs assigned the death penalty more than those who saw sadness in VISs (Nuñez et al., 2017). But judges perceived anger in VISs as less acceptable than other emotions (Schuster and Propen, 2010). As for positive moral emotions, one experiment examined the role of empathy and sympathy for a victim's family and found that it mediated the effects of VISs on death penalty decisions—with greater empathy/sympathy predicting more death penalty sentencing (Paternoster and Deise, 2011). The Kunst et al. (2021) meta-analysis separately investigated the effects of harm to the victim, victim affect, and the emotional content expressed in decision-making, finding inconsistent effects. Taking a broader view and considering the overall impact (including the severity of crime, the type of harm, and emotional content), research findings suggest that the overall impact of the crime on the victim should influence juror decision-making.

### Perspective taking

Perspective taking is described as a “cognitive” aspect of empathy, involving imagining the thoughts and feelings of another person, whereas empathy or empathic concern involves feeling with or for others (Batson, 2010; Hodges and Wegner, 1997). Although theorized as distinct, evidence suggests that taking others' perspectives can also influence the way individuals feel about those others, influencing how they are perceived and treated (Archer et al., 1979; Batson et al., 2005), increasing forgiveness (McCullough et al., 1997), and helping reduce conflict (Franzoi et al., 1985; Galinsky et al., 2008; Takaku et al., 2002).

One reason perspective taking has these effects is because it sometimes prompts perspective takers to see themselves as more similar to targets, which may involve seeing the target as more “self-like” or seeing oneself as more “target-like” (Davis et al., 1996; Hodges et al., 2011; Galinsky et al., 2005, 2008; Galinsky and Moskowitz, 2000; Goldstein and Cialdini, 2007; Laurent and Myers, 2011; Skorinko et al., 2012). This perceived overlap can be a driving force in changing perspective takers' beliefs about themselves and others (Laurent and Myers, 2011; Myers et al., 2014) and may also predict whether perspective takers engage in helping behaviors (Cialdini et al., 1997; Maner et al., 2002).

Perspective taking, however, is not always consistent. Perspective takers may anchor onto available information, may not be able to sufficiently adjust away from their own egocentric perspectives, or may see more differences than similarities between themselves and perspective-taking targets (Epley et al., 2004; Frantz and Janoff-Bulman, 2000; Hodges et al., 2018; Skorinko and Sinclair, 2013). The target of the perspective-taking endeavor also matters. For instance, taking the perspective of a defendant leads to *decreased* perceptions of culpability, whereas taking the perspective of a victim leads to *increased* perceptions of culpability (Skorinko et al., 2014). Likewise, after taking a victim's perspective, participants place greater blame on the perpetrator than on the imagined victim (Catellani and Milesi, 2001).

### Present research

The current work extends past work on VISs by examining the effects of the perspective-taking target and the impact of crime on victim(s) stated in VISs on mock juror perceptions of guilt/sentencing, culpability, and recidivism. In four experiments, we investigate the simultaneous effects of perspective taking and VISs on perceptions of criminal defendants and related variables (e.g., empathy).

In Experiment 1, we investigate whether the target of perspective taking (defendant, victim, both, or none) and the impact of the crime described in the VISs (low or high) influences perceptions of the defendant. We hypothesize that the impact of the crime on the victim expressed in the VISs will influence juror decision-making, such that high-impact VISs will result in more negative perceptions of the defendant in terms of guilt, sentencing, culpability, and recidivism. We also hypothesize that taking the perspective of the victim(s), especially when presented with high-impact VISs, should lead to more negative perceptions of the defendant. However, based on past research (Skorinko et al., 2014), we predict the opposite effect to occur when jurors take the defendant's perspective, at least when the impact of the crime is low. Because the courtroom is a dynamic environment, it is also possible that jurors could be prompted to perspective take with both the defendant and the victim. Thus, we also explore this possibility; however, we have no *a priori* predictions of the outcome as, from past work, what might occur is unclear.

Experiment 2 expands on this to test whether the effects replicate in a non-capital case (armed robbery). Because past research using non-capital cases has found some evidence that the impact of the crime in VISs matters (Nadler and Rose, 2003; Wevodau et al., 2014a), we hypothesize a main effect for VISs and an interaction between the perspective-taking target and the VIS on perceptions of the defendant. In Experiment 3, we again use a capital murder case and investigate whether the type of perspective taking (imagining the self vs. imagining the other) influences juror decision-making. Past research has found that imagining the other can cue self-evaluative thoughts (Vorauer and Sasaki, 2014; see Experiment 3 for more discussion). Therefore, we hypothesize that those who imagine the other (i.e., the defendant) will perceive the defendant negatively, especially when the VIS's impact is high. However, we hypothesize that those who imagine the self will be able to perceive the defendant more favorably, especially when the VIS's impact is low, because this type of perspective taking does not cue self-evaluation. Finally, although Experiments 1–3 prompt jurors to perspective take through instructions by an attorney, it is also possible that jurors may engage in perspective taking without any instructions. Therefore, in Experiment 4, we examine whether priming a mindset to take the defendant's perspective works differently from explicit instructions to take the defendant's perspective. We hypothesized that participants primed to take the defendant's perspective would perceive the defendant more favorably when presented with a low-impact VIS but more negatively when presented with a high-impact VIS.

## Experiment 1

Experiment 1 investigates whether the target of perspective taking (defendant, victim, both, or none) and the impact of the crime on victims (low- or high-impact VIS) influence mock jurors' decisions and empathy. Based on past research (Myers et al., 2018; Skorinko et al., 2014), we hypothesize that when low-impact VISs are presented, perspective taking with the defendant will lead to more favorable perceptions of the defendant but that this will be less likely when high-impact VISs are presented. Likewise, we hypothesize that perspective taking with the victim will lead to more negative perceptions of the defendant. Because courtrooms are dynamic environments, we also explore what happens when perspective-taking instructions focus on both victims and defendants. We have no *a priori* predictions as past work has not investigated perspective taking with multiple targets in this context.

## Method

### Participants

A total of 208 individuals (55% female, 44% male, and 1% did not disclose) participated in this online experiment. All participants provided informed consent and were debriefed. Participants were from online participant platforms^4^ (40% Amazon's Mechanical Turk and 40% SocialSci) and a participant pool from a university in the northeastern United States (21%). Those participating through an online platform were paid ($0.50 MTurk and $2 SocialSci), and those from the university participant pool earned course credit. The computer program randomly assigned participants to each condition with the goal of keeping cell sizes as even as possible. Participants mostly self-identified as White (72%; 11% Asian, 6% Black, 4% Latinx, 1% Other, and 6% did not disclose), and ranged in age from 17^5^ to 72 (*M* = 31.46, *SD* = 13.64). Most participants were U.S. citizens (90%), not currently engaged in higher education (60% not students, 36% university students, and 3% did not disclose), and more likely to be politically^6^ moderate (58%) than conservative (26%) or liberal (13%). Participants were not biased toward either the defense or the prosecution (*M* = 4.28, *SD* = 0.91) on the Juror Bias Scale^7^ (Kassin and Wrightsman, 1983). In all, 27 participants were removed from analyses (1 reported being 15 years old, 21 were not U.S. citizens or did not disclose citizenship, 4 spent 2 min or less on the study, and 1 spent less 5 min and failed to respond to multiple measures), leaving a final sample size of 181 participants.^8^

### Design and materials

The experiment was a 4 (Perspective Taking: no perspective taking, perspective taking with the defendant, perspective taking with the victim, or perspective taking both) × 2 (VIS: low impact vs. high impact) between participant design. Participants were randomly assigned to take the perspective with (a) the defendant, (b) the victim, (c) both the defendant and the victim, or (d) no one (no perspective taking control condition). Perspective taking was prompted by the attorneys in their closing arguments (e.g., the defense attorney prompted perspective taking with the defendant).

Participants also read two VISs (one by the victim's fiancée and one by the victim's father) that either did not express much impact in terms of what happened to them as a result of the crime (low impact) or clearly expressed the impacts of the crime on their lives (high impact). We chose to look at the overall impact because some past work has found that the impact of the crime (mild or severe) expressed in the VIS influences death penalty decisions (Mitchell et al., 2016; Myers et al., 2002). In the low-impact condition, the fiancée stated that they were planning to get married but provided no additional details, and the victim's father stated that he was devastated and had difficulty keeping up at work. In the high-impact condition, the fiancée articulated that the murder destroyed her plans for a future, and the father discusses the impacts it had on his and his wife's work and their mental health and expresses how he will never get over the death. See Appendix A for the perspective-taking prompts and Appendix B for the VIS statements.

### Measures

#### Sentencing decision

In all conditions, the defendant was guilty of murder. Participants, acting as jurors, decided whether the defendant should receive the death penalty or life in prison without parole.

#### Culpability and recidivism

Although this was a sentencing trial, we were still interested in whether the target of perspective taking and impact expressed in VISs would impact perceptions of culpability and recidivism as these factors may influence death penalty decisions. Participants indicated to what extent the defendant (a) was at fault, (b) would commit a similar crime in the future, (c) was blameworthy, and (d) was responsible for the crime on a 7-point Likert-type scales (1 = *Not at all*; 7 = *Very much*). As in past research (Skorinko et al., 2014), we analyzed culpability (i.e., fault, blame, and responsibility) separately from recidivism (i.e., commit a similar crime in the future). The fault, blame, and responsibility items were averaged to create the culpability measure (α = 0.79), and the single item regarding committing a similar crime in the future measured recidivism.

#### Empathy with the defendant, the victim, and the victim's family

Participants indicated their empathy, sympathy, compassion, softheartedness, warmth, tenderness, and how moved they felt toward the defendant, the victim, and the victim's family (see Batson et al., 1997) on a 7-point scale (1 = *Not at all*; 7 = *Very much*). These seven items were aggregated into single measures of empathy for the defendant (α = 0.92), the victim (α = 0.94), and the victim's family (fiancée and father, α = 0.96).^9^

#### VIS emotionality and ability to perspective take

Participants rated how emotional the VIS testimony of the fiancée and father was on a 7-point scale (1 = *Not at all*; 7 = *Very much*). These two items were averaged together (*r* = 0.55). Participants also indicated how easy it was and how motivated they were to put themselves in the shoes of the defendant, the victim, the victim's fiancée, and the victim's father on a 7-point scale (1 = *Not at all*; 7 = *Very much*). Separate measures for the ability to perspective take were created for the defendant (*r* = 0.69), the victim (*r* = 0.74), and the victim's family (α = 0.85).

#### Demographics and exploratory items

Participants provided information about their gender identity, citizenship, age, ethnicity/race, if they were enrolled in university, political orientation, and if they had a recent death in their family. Additional exploratory items were assessed because these measures were important factors in past work in either VIS or perspective-taking studies (e.g., self–other overlap and the Interpersonal Reactivity Index). Because they were not central to the main hypotheses and were not statistically significant predictors or mediators, these items are not described in the text but are available in Appendix C.

### Procedure

After providing informed consent, participants read a summary transcript of a capital murder trial where the defendant was convicted of first-degree murder. The summary also included a transcript of the penalty phase. In this section, participants were randomly assigned to read closing arguments with no perspective-taking prompts (control condition) or prompts from the prosecuting attorney, defense attorney, or both to respectively take the perspective of the victim, the defendant, or both. Following this, participants were randomly assigned to read two VIS transcripts—one from the victim's fiancée and one from the victim's father. Half the participants read two statements that articulated the high impact of the crime on the victims, and the remaining half read two statements that were low in impact. Participants then rendered a sentence (i.e., death penalty or life in prison) and provided their perceptions of and empathy toward the defendant and the victims. After providing demographic information, participants were thanked, viewed an online debriefing document, and were awarded payment or credit.

## Results

We conducted separate 2 (VIS: low vs. high impact) × 4 (Perspective Taking: no instruction, perspective taking defendant, perspective taking victim, perspective taking both) analyses of variance (ANOVAs) on each dependent measure. The exception to this was for sentencing, which was examined using a generalized linear model that specified a binary logistic outcome to account for participants' choice of life in prison vs. death for the defendant.^10^

### Sentencing

More people believed the defendant should be sentenced to life in prison (*n* = 133) than sentenced to death (*n* = 47). There were no effects of VIS (*p* = 0.568), perspective taking (*p* = 0.527), or the interaction between VIS and perspective taking (*p* = 0.308) on sentencing decisions.

### Culpability and recidivism for defendant

**Figure 1:**
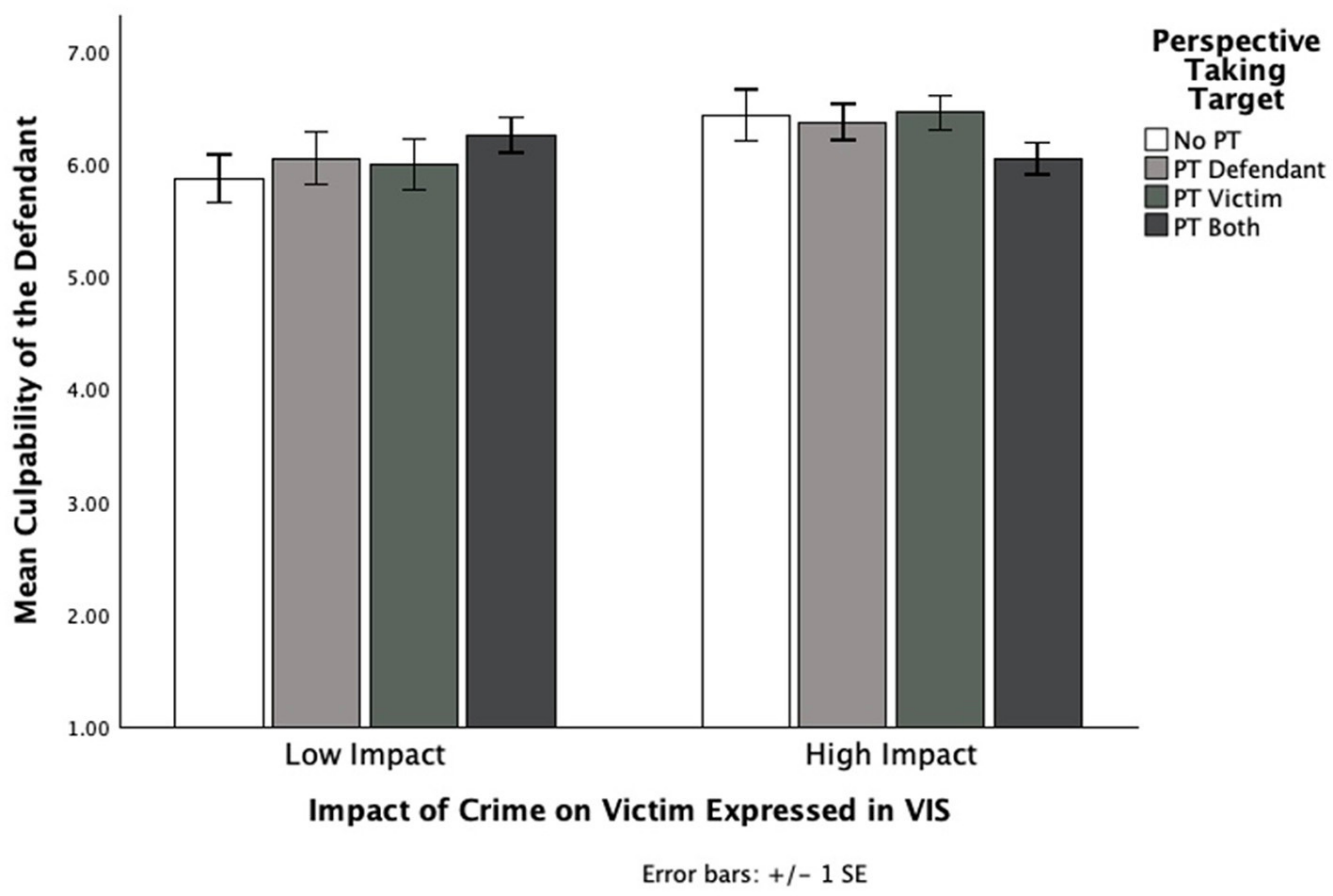
The effects of impact of crime on victim VIS and perspective taking on perceived culpability of a defendant in Experiment 1. PT means perspective taking. VIS means victim impact statement. The main effect for the impact of VIS was statistically significant with high-impact VIS leading to greater culpability than low-impact VIS, *p* = 0.038. There was no main effect for perspective taking (*p* = 0.971), nor was there a significant interaction between VIS and perspective taking (*p* = 0.170).

There was a main effect for VIS, *F*_(1, 173)_ = 4.376, *p* = 0.038, $\eta_{\text{p}}^{2}$ = 0.025, 95% CI (0.02, 0.55). As seen in Figure 1, those who saw the high-impact VIS (*M* = 6.31, *SD* = 0.80) believed the defendant was more culpable than those who saw the low-impact VIS (*M* = 6.05, *SD* = 0.99), *F*_(1, 173)_ = 4.376, *p* = 0.038, $\eta_{\text{p}}^{2}$ = 0.025, 95% CI (0.02, 0.55). Figure 1 also shows that there was no main effect for perspective taking (*p* = 0.971), nor was there a significant interaction between VIS and perspective taking (*p* = 0.170). For recidivism, there were no main effects for VIS (*p* = 0.892), perspective taking (*p* = 0.318), or any interaction (*p* = 0.091).

### Empathy for defendant and victim^11^

There were no effects of VIS (*p* = 0.153), perspective taking (*p* = 0.241), or of their interaction (*p* = 0.139) on empathy for the defendant. There were also no effects of VIS (*p* = 0.089) or perspective taking (*p* = 0.544) or any interaction between VIS and perspective taking (*p* = 0.690) on empathy for the victim.

VIS had a small effect on empathy for the victim's family, *F*_(1, 173)_ = 7.111, *p* = 0.008, $\eta_{\text{p}}^{2}$ = 0.04, 95% CI (0.14, 0.90). Participants in the high-impact condition (*M* = 5.45, *SD* = 1.25) reported more empathy for the family than those in the low-impact condition (*M* = 4.94, *SD* = 1.37). There was also an effect of perspective taking, *F*_(1, 173)_ = 2.944, *p* = 0.034, $\eta_{\text{p}}^{2}$ = 0.05. Those who took the perspective of the defendant felt the least amount of empathy toward the victim's family (*M* = 4.77, *SD* = 1.63). There was no interaction between VIS and perspective taking, *p* = 0.766.

### VIS emotionality and ability to perspective take

Confirming the manipulation, participants perceived the high-impact VIS (*M* = 5.50, *SD* = 1.40) as being more emotional than the low-impact VIS (*M* = 4.41, *SD* = 1.44), *F*_(1, 173)_ = 28.52, *p* < 0.001, $\eta_{\text{p}}^{2}$ = 0.142, 95% CI (0.71, 1.55). No effects of perspective taking (*p* = 0.307) or the interaction between VIS and perspective taking (*p* = 0.208) were observed.

Regarding the ability to perspective take with the defendant, there were no main effects of VIS (*p* = 0.797) or perspective taking (*p* = 0.155). However, there was an interaction between perspective taking and VIS, *F*_(3, 173)_ = 4.19, *p* = 0.007, $\eta_{\text{p}}^{2}$ = 0.07. In the low-impact-VIS condition, the omnibus effect of perspective taking was not significant, *p* = 0.306. In the high-impact-VIS condition, however, the omnibus effect of perspective taking was significant, *p* = 0.005. The reported ability to take the defendant's perspective was highest in the perspective taking for both conditions (*M* = 4.80, *SD* = 1.08). The next highest was in the no-perspective-taking condition (*M* = 3.41, *SD* = 1.99) and the perspective-taking-defendant condition (*M* = 3.38, *SD* = 1.85). The lowest was in the perspective-taking-victim condition (*M* = 3.25, *SD* = 1.86). *Post-hoc* tests showed that the perspective-taking ability in the perspective taking for both conditions was significantly higher (*p*s < 0.042) than all other conditions, which did not differ from each other, *p*s > 0.991.

For the ability to take the perspective of the victim, there was no interaction between VIS and perspective taking (*p* = 0.834) or a main effect for perspective taking (*p* =0.272). However, VIS was a significant predictor, with a higher reported ability to take the victim's perspective in the high-impact (*M* = 5.41, *SD* = 1.39) than the low-impact condition (*M* = 4.91, *SD* = 1.57), *F*_(1, 173)_ = 5.31, *p* = 0.022, $\eta_{\text{p}}^{2}$ = 0.03, 95% CI (0.07, 0.96).

We also looked at the ability to perspective take with the victim's family. There was no interaction between VIS and perspective taking (*p* = 0.610). VIS was a significant predictor, with greater reported ability to perspective take in the high-impact (*M* = 4.95, *SD* = 1.44) relative to the low-impact-VIS condition (*M* = 4.45, *SD* = 1.47), *F*_(1, 173)_ = 5.88, *p* = 0.016, $\eta_{\text{p}}^{2}$ = 0.03, 95% CI (0.10, 0.94). There was also an omnibus effect of perspective taking, *F*_(3, 173)_ = 3.15, *p* = 0.026, $\eta_{\text{p}}^{2}$ = 0.05. Those who took the perspective of the defendant reported the lowest ability to perspective take with the victim's family (*M* = 4.11, *SD* = 1.67). *Post-hoc* Tukey tests revealed that the ability to perspective take with the victim tended to be lower in the perspective-taking-defendant condition (*M* = 4.11, *SD* = 1.67) than in the no-perspective-taking condition (*M* = 5.03, *SD* = 1.49), *t*_(173)_ = 2.94, *p* = 0.019, 95% CI (0.11, 1.72). No other effects were significant (*p*s > 0.099), but the pattern was the same as those taking the perspective of the defendant tended to report a lower ability to perspective take with the victim's family.

## Summary

Overall, participants assigned the life in prison sentence more than the death penalty, and neither VIS nor perspective taking influenced these decisions. VIS did influence culpability. Those who saw high impact (vs. low) VIS found the defendant more culpable. VIS also had a small significant effect on empathy felt for the victim's family. This result suggests that VISs influences empathic concern for family members impacted by a crime.

Perspective taking did not have any effects on juror decisions or empathy. However, there was an interaction between perspective taking and VIS on the reported ability to perspective take with the defendant. When the impact of the crime was high, those who took the perspective of both the defendant and the victim reported the highest ability to take the defendant's perspective, while those who took the victim's perspective reported the least ability. Although this finding suggests an impact of the perspective-taking manipulation on the reported ability to take the defendant's perspective, it is also possible that this result represents a Type 1 error. That is, the finding for perspective taking only emerged in the VIS condition, and decomposition of the effect showed that the reported ability to take the defendant's perspective was highest in the perspective taking with both conditions relative to all other conditions (including the perspective-taking-defendant condition). Speculatively, if this was not a Type 1 error, one might consider participants' natural inclination to take the perspective of the victim when the impact of the crime is high (Myers et al., 2018). However, being asked to consider the perspectives of both the victim and defendant may have licensed participants' ability to also consider the defendant's perspective. Meanwhile, only asking them to consider the defendant's perspective might have led to difficulty in doing so, and providing no instruction or asking them to take the victim's perspective may have reinforced their natural inclination. Future research using a similar design might clarify these issues.

Perhaps the most notable finding was that the higher (vs. lower) impact VISs led to participants viewing the defendant as more culpable and feeling more empathy toward the victim's family. Perspective taking, in contrast, had limited effects. The lack of effects for perspective taking were possibly due to this being a death penalty case. Consistent with this idea, only 29% of previous experiments using death penalty cases found effects for VIS, whereas 71% of experiments using non-capital cases found effects for VIS (Kunst et al., 2021). Thus, in Experiment 2, we examine the effects of perspective taking and VISs in a non-capital case.

## Experiment 2

In Experiment 1, VISs that expressed higher (vs. lower) impact on victims led to increased perceptions of culpability, feelings of empathy with the victim's family, and higher reported ease of taking the victim's family's perspective. Experiment 1 relied on a capital case because this is one instance in which VISs are allowed and jurors can make sentencing decisions (e.g., the death penalty). Using this case type is consistent with past work on VIS (Kunst et al., 2021; Wevodau et al., 2014a). However, the rate of VIS being presented in non-capital cases has increased (Wevodau et al., 2014a; Logan, 1999), and although rare, juries contribute to sentencing in some jurisdictions. Thus, understanding how VISs affect juror perceptions in non-capital cases is important. In the limited research using non-capital cases, mock jurors exposed to VISs tend to be more punitive toward the defendant than those not exposed (Nadler and Rose, 2003; Tsoudis and Smith-Lovin, 1998; Wevodau et al., 2014a,b). Regarding the impact of the crime on the victim, one study found that harsher sentences were given when VISs described severe compared to mild emotional injuries for robbery, but there were no differences between severe VIS and no VIS conditions (Nadler and Rose, 2003). In another study, participants recommended harsher sentences when VIS were presented (vs. not) for sexual assault, regardless of the VIS expressing emotional content or harm to the victim (Wevodau et al., 2014a).

Experiment 2 expands on this past work by investigating the effects of the perspective-taking target (defendant, victim, or neither) and the overall impact of the crime on the victim (high or low) in a non-capital case (armed robbery). Given past findings, we hypothesize a main effect for VIS and an interaction between the perspective-taking target and the VIS on perceptions of the defendant. More specifically, we predict that perspective taking with the victim will result in more negative perceptions of the defendant, especially in the high-impact-VIS condition. However, we hypothesize that taking the defendant's perspective will lead to more favorable perceptions of the defendant but only in the low-impact-VIS condition.

## Method

### Participants

A total of 248 individuals (44% female; 55% male, 1% other) participated in this online experiment. All participants provided informed consent and were debriefed. Participants came from Amazon's Mechanical Turk (72%) and a participant pool from a university in the northeastern United States (28%). Students received course credit, and Mechanical Turk participants were paid $3. Participants were randomly assigned to each condition with the goal of keeping cell sizes as even as possible. Among participants, 74% identified as White (8% Asian, 7% Black, 5% Latinx, 1% Middle Eastern, 1% Native American, 4% Multiracial or Other, and 1% did not disclose) and ranged in age from 18 to 71 (*M* = 35.82, *SD* = 13.90). Most participants were citizens of the United States (98%), not engaged in higher education (62% not students, 38% university students, and 1% did not disclose), politically liberal (49%^12^), and not biased toward the defense or the prosecution (*M* = 4.4, *SD* = 0.86) on the Juror Bias Scale (Kassin and Wrightsman, 1983). In total, 15 participants were removed from the analyses (six were not U.S. citizens, three thought it was a murder case, one indicated they did not read the trial, four were likely bots based on open-ended responses, and one was under 17 with no parental consent). The analyses presented here are based on 233 participants.

### Design, materials, and procedure

The design of Experiment 2 was the same as in Experiment 1 except for a few key factors (a) the victim was injured (not murdered) during an armed robbery, (b) the VIS was made by the victim (not their family) and was modified to reflect injuries sustained during an armed robbery (see Appendix D), (c) the perspective taking with both the defendant and victim condition was removed, and (d) participants rendered a verdict and determined a sentence for first-degree armed robbery.

After giving informed consent, participants read about an armed robbery case in which the victim was shot but not killed. In the closing arguments, participants were randomly assigned to be prompted to perspective take with the victim, the defendant, or received no perspective-taking prompt. Participants then read a single VIS from the victim that expressed either low or high impact. In the low-impact condition, the victim stated what happened during the armed robbery and that they now suffer from Post Traumatic Stress Disorder (PTSD) and have not returned to work or school. In the high-impact condition, the victim stated what happened to them but elaborated, with details, about thinking that they were going to die, the pain and fear they still have, and how their medical career has been put on hold. As this was a non-capital case, participants gave a verdict. If they found the defendant guilty, they rendered a sentence from 0 to 30 years (after seeing federal sentencing guidelines of 10–20 years for armed robbery). Participants also indicated the culpability of the defendant (α = 0.98), empathy toward the defendant (α = 0.98), empathy toward the victim (α = 0.95), emotionality of the VIS, and perspective-taking ability with the defendant (*r* = 0.74) and the victim (*r* = 0.72). Participants provided demographic information including gender, citizenship, age, level of education, ethnicity, political orientation, and whether they had served on a jury or knew any victims of robbery. Participants were then thanked, debriefed online, and awarded payment/credit.

## Results

Participants' responses to the verdict measure, which was dichotomous, were analyzed using a generalized linear model that specified a binary logistic outcome. Other variables were examined using a 2 (VIS: high vs. low impact) × 3 (Perspective Taking: control, perspective taking with the defendant, or perspective taking with the victim) ANOVAs.

### Verdict and sentencing

Overall, participants were more likely to render a not guilty verdict (*n* = 147) than a guilty verdict (*n* = 84), χ^2^ (1, *N* = 233) = 18.13 *p* < 0.001. The VIS impacted verdicts, with more people in the high-impact-VIS condition (*n* = 53) finding the defendant guilty than in the low-impact-VIS condition (*n* = 31) and more people in the low-impact-VIS condition (*n* = 85) finding the defendant not guilty than in the high-impact-VIS condition (*n* = 62), Wald χ^2^ = 8.97, *p* = 0.003, ϕ = 0.20. Perspective taking was not a significant predictor (*p* = 0.925), nor was the interaction between perspective taking and VIS, *p* = 0.352. Of the participants who made sentencing decisions (*n* = 84), there were no effects for VIS, perspective taking, or the interaction between perspective taking and VIS, *p*s > 0.177.

### Culpability of defendant and recidivism

**Figure 2:**
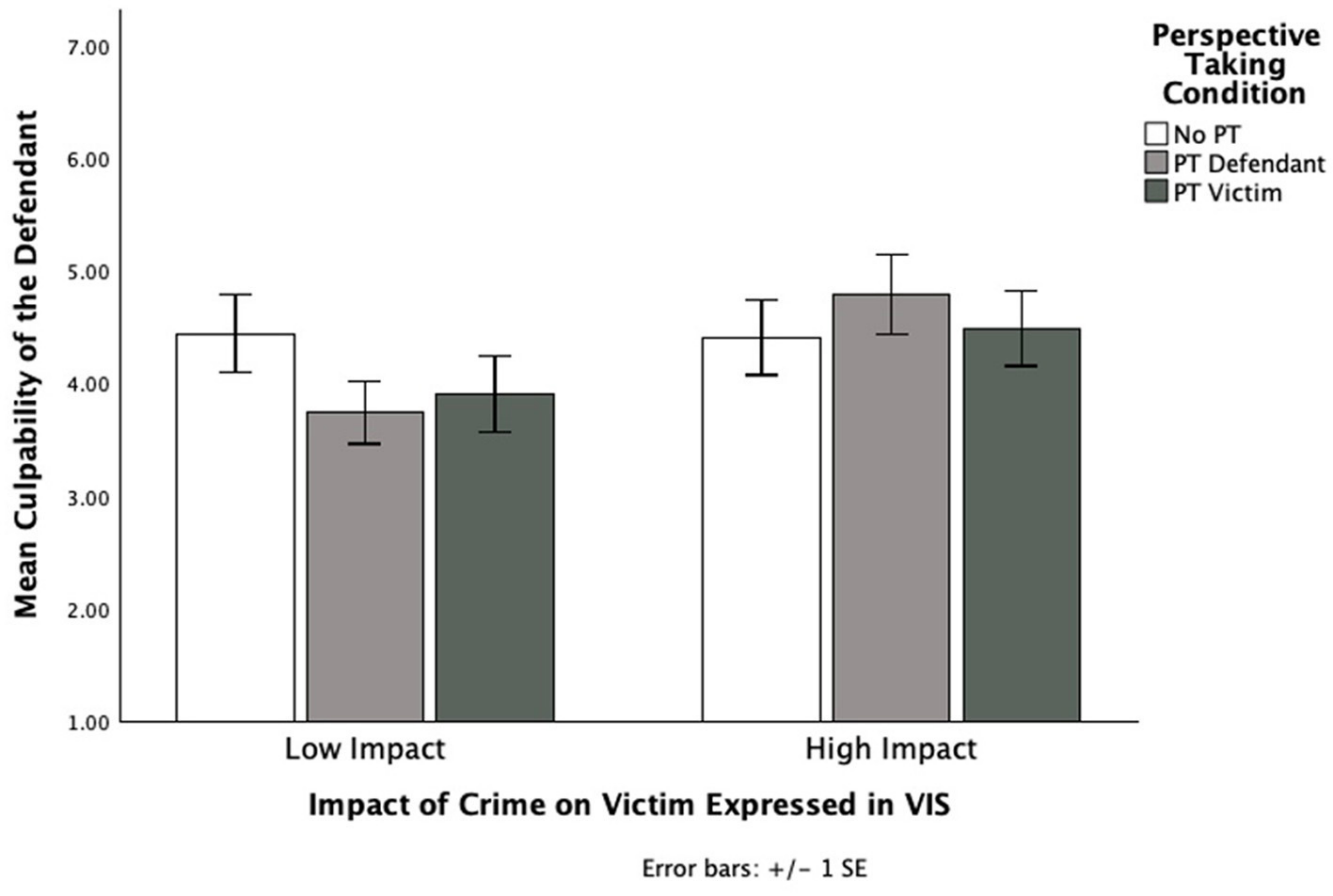
The effects of impact of crime on victim VIS and perspective taking on perceived culpability of a defendant in Experiment 2. PT means perspective taking. VIS means victim impact statement. The main effect for the impact of VIS was statistically significant, with high-impact VIS leading to greater culpability than low-VIS, *p* = 0.049. There was no main effect for perspective taking (*p* = 0.788) or interaction between VIS and perspective taking (*p* = 0.262).

As in Experiment 1, there was a main effect for VIS, *F*_(1, 226)_ = 3.912, *p* = 0.049, $\eta_{\text{p}}^{2}$ = 0.017, 95% CI (0.00, 1.07). As seen in Figure 2, participants who saw the high-impact VIS (*M* = 4.56, *SD* = 2.10) believed the defendant was more culpable than those who saw the low-impact VIS (*M* = 4.01, *SD* = 2.01). There was no main effect for perspective taking (*p* = 0.788) or interaction between VIS and perspective taking (*p* = 0.262; see Figure 2). For recidivism, there was a significant main effect for VIS such that those exposed to the high-impact VIS (*M* = 4.03, *SD* = 1.83) thought the defendant would recidivate more than those exposed to the low-impact VIS did (*M* = 3.56, *SD* = 1.70), *F*_(1, 225)_ =3.924 *p* = 0.049, $\eta_{\text{p}}^{2}$ = 0.017, 95% CI (0.00, 0.93). There was no effect for perspective taking (*p* = 0.688) or interaction (*p* = 0.772).

### Empathy for defendant and victim

As in Experiment 1, there were no effects of perspective taking (*p* = 0.573) and no interaction between VIS and perspective taking (*p* = 0.500) on empathy for the defendant. There was a main effect for VIS, such that those exposed to low-impact VIS (*M* = 3.73, *SD* = 1.77) expressed more empathy for the defendant than those exposed to high-impact VIS (*M* = 3.04, *SD* = 1.68), *F*_(1, 227)_ = 9.002, *p* = 0.003, $\eta_{\text{p}}^{2}$ = 0.038, 95% CI (0.23, 1.13). Similar to Experiment 1, there were no effects of VIS, perspective taking, or their interaction for empathy toward the victim (*ps* > 0.371).

### VIS emotionality and ability to perspective take

As expected, but with a relatively small effect size, participants regarded emotionality as higher in the high-impact (*M* = 5.00, *SD* = 1.44) vs. the low-impact (*M* = 4.56, *SD* = 1.40) VIS, *F*_(1, 226)_ = 5.39, *p* = 0.021, $\eta_{\text{p}}^{2}$ = 0.021, 95% CI (0.07, 0.81). The effects of perspective taking and the interaction between the two variables were not significant, *p*s > 0.745.

For perspective-taking ability with the defendant, the only significant effect was for VIS (other *p*s > 0.933). Participants reported a greater ability to take the defendant's perspective in the low-impact (*M* = 4.43, *SD* = 1.78) relative to the high-impact VIS (*M* = 3.64, *SD* = 1.78), *F*_(1, 227)_ = 11.48, *p* < 0.001, $\eta_{\text{p}}^{2}$ = 0.048, 95% CI (0.33, 1.26). But neither VIS, perspective taking, nor the interaction between VIS and perspective taking predicted the ability to perspective take with the victim, all *p*s > 0.568.

## Summary

The results of Experiment 2 were like those of Experiment 1. The VIS influenced perceptions of culpability, with greater culpability in the high- vs. low-impact condition. The VIS also influenced guilt decisions, with more people believing the defendant was guilty in the high-impact (vs. low-impact) condition. While low-impact (vs. high-impact) VIS predicted greater empathy with the defendant, this did not occur in Experiment 1. The results from Experiments 1 and 2 suggest that the type of case (capital vs. non-capital) does not matter for perceptions of culpability. However, case type may matter for empathy felt for the defendant because participants felt more empathy for the defendant when the VIS's impact was low compared to high in a non-capital case (Experiment 2), but this finding did not emerge in a capital case (Experiment 1). Unlike Experiment 1, where perspective taking and VIS interacted to predict participants' ability to take the defendant's perspective, only VIS had an impact on perspective-taking ability in Experiment 2 (i.e., greater perspective-taking ability when the VIS was low impact rather than high impact). The crime type may possibly help explain this, as Experiment 1 used capital murder and Experiment 2 did not. In Experiment 3, we examine whether the type of perspective taking (self vs. other) with the defendant matters.

## Experiment 3

Experiment 3 took a different approach and examined whether differences in the type of perspective taking influenced decision-making. Specifically, participants either imagined *themselves as* the defendant (perspective-taking self) or imagined what the defendant was going through (perspective-taking other; see Batson et al., 1997; Myers et al., 2014; Vorauer and Sasaki, 2014). We used this method because defendants may be at a disadvantage as they are accused of a crime that a juror may not be able to imagine doing. Moreover, imagining the self during perspective taking can increase empathy and influence motivations to help but may also increase personal distress, whereas imagining the other increases empathy (e.g., Batson et al., 1997; Myers et al., 2014). However, imagining the other cues self-evaluation, which is not the case when imagining the self (Vorauer and Sasaki, 2014). Because imagining the other can cue self-evaluation, we hypothesize that those who imagine the other (the defendant in this case) will perceive the defendant negatively, especially when the VIS impact is high. We also hypothesize that those who imagine the self will perceive the defendant more favorably, especially when the VIS's impact is low. However, given the results of Experiments 1 and 2, where few effects of perspective taking emerged, having a strong prediction about its effects is difficult.

## Method

### Participants

A total of 255 individuals (50% female, 49% male, and 1% transgender) participated in this online experiment. All participants provided informed consent and were debriefed. Participants came from an online platform (76% SocialSci) and a university in the northeastern United States (24%). Those participating through an online platform were paid ($2) and those participating through the university earned course credit. Participants were randomly assigned to the conditions with the goal of keeping cell sizes as even as possible. Among participants, 74% identified as White (8% Asian, 6% Black, 6% Latinx, 3% Multiracial or Other, and 3% did not disclose), and ranged in age from 17^13^ to 75 (*M* = 25.57, *SD* = 10.28). Participants were U.S. citizens (93%), and more than two thirds were engaged in higher education (32% not students, 67% university students, and 1% did not disclose). Participants were not biased toward the defense or the prosecution (*M* = 4.1, *SD* = 0.80; Kassin and Wrightsman, 1983). In total, 27 participants were removed from the analyses (17 were not U.S. citizens, and 10 completed the study in 3 min or less). The analyses are based on 227 participants.

### Design, materials, and procedure

Participants were randomly assigned to one cell of a 3 (Perspective Taking: no perspective taking, imagine oneself as the defendant, imagine defendant) × 2 (VIS: high vs. low impact) between participant design. For the perspective-taking manipulation, participants either received no perspective-taking instructions (control), were instructed to imagine they were the defendant (perspective-taking self), or were instructed to imagine what the defendant was going through (perspective-taking other). For the VIS manipulation, participants were randomly assigned to read two VISs that were either high or low in impact (the same VISs as in Experiment 1).

After giving informed consent, participants read details of a capital murder trial. As part of the defense's closing arguments, participants were randomly assigned to imagine the self as the defendant (perspective-taking self), imagine the defendant (perspective-taking other), or received no perspective-taking prompt (control condition). Adapting methods used in past research (Batson et al., 1997; Vorauer and Sasaki, 2014), those in the perspective-taking-self condition were instructed to imagine *themselves* as if they were the defendant in the case. In other words, they were asked to take a first-person point of view. Participants in the perspective-taking-other condition were instead instructed to imagine the defendant and the defendant's position (i.e., a third-person point of view). Thus, the focal point of the perspective taking differed: focus on self vs. focus on the defendant. Although this difference is subtle, it has been effective in past research (see Vorauer and Sasaki, 2014).

Participants then read a VIS by the victim's fiancée and another by the victim's father. Both statements were either high or low in impact of the crime on the victims. Participants then determined a sentence (death penalty or life in prison) and indicated the culpability of the defendant (α = 0.83), empathy with the defendant (α = 0.94), empathy with the victim (α = 0.95), and empathy with the victim's fiancée^14^ (α = 0.94). Participants also reported how emotional they found the fiancée's VIS to be, and their ability to perspective take with the defendant,^15^ the victim (*r* = 0.73), and the victim's fiancée (*r* = 0.73). Participants provided demographic information including gender, citizenship, age, level of education, ethnicity, and whether they had a recent death in their family. Then participants were thanked, debriefed, and awarded payment or credit.

## Results

To account for the dichotomous nature of sentencing, this variable was analyzed using a generalized linear model that specified a binary logistic outcome. Other dependent measures were analyzed using 2 (VIS: low vs. high impact) × 3 [Perspective Taking: no perspective taking, perspective-taking self (imagine oneself), perspective-taking other (imagine defendant)] ANOVAs.

### Sentence

More participants thought the defendant should be sentenced to life in prison (*n* = 143) than death (*n* = 84), χ^2^ (1, *N* = 227) = 15.34, *p* < 0.001. There was an effect for VIS on sentencing. More participants (*n* = 52) believed the victim should be put to death in the high-impact condition (low impact, *n* = 32), but relatively equal numbers of participants (*n* = 70 in high-impact VIS; *n* = 73 in low-impact VIS) selected life in prison, Wald χ^2^ = 4.53, *p* = 0.034, ϕ = 0.13. The perspective-taking manipulation and interaction between perspective taking and VIS were not significant, *p*s > 0.307.

### Culpability of defendant and recidivism

**Figure 3:**
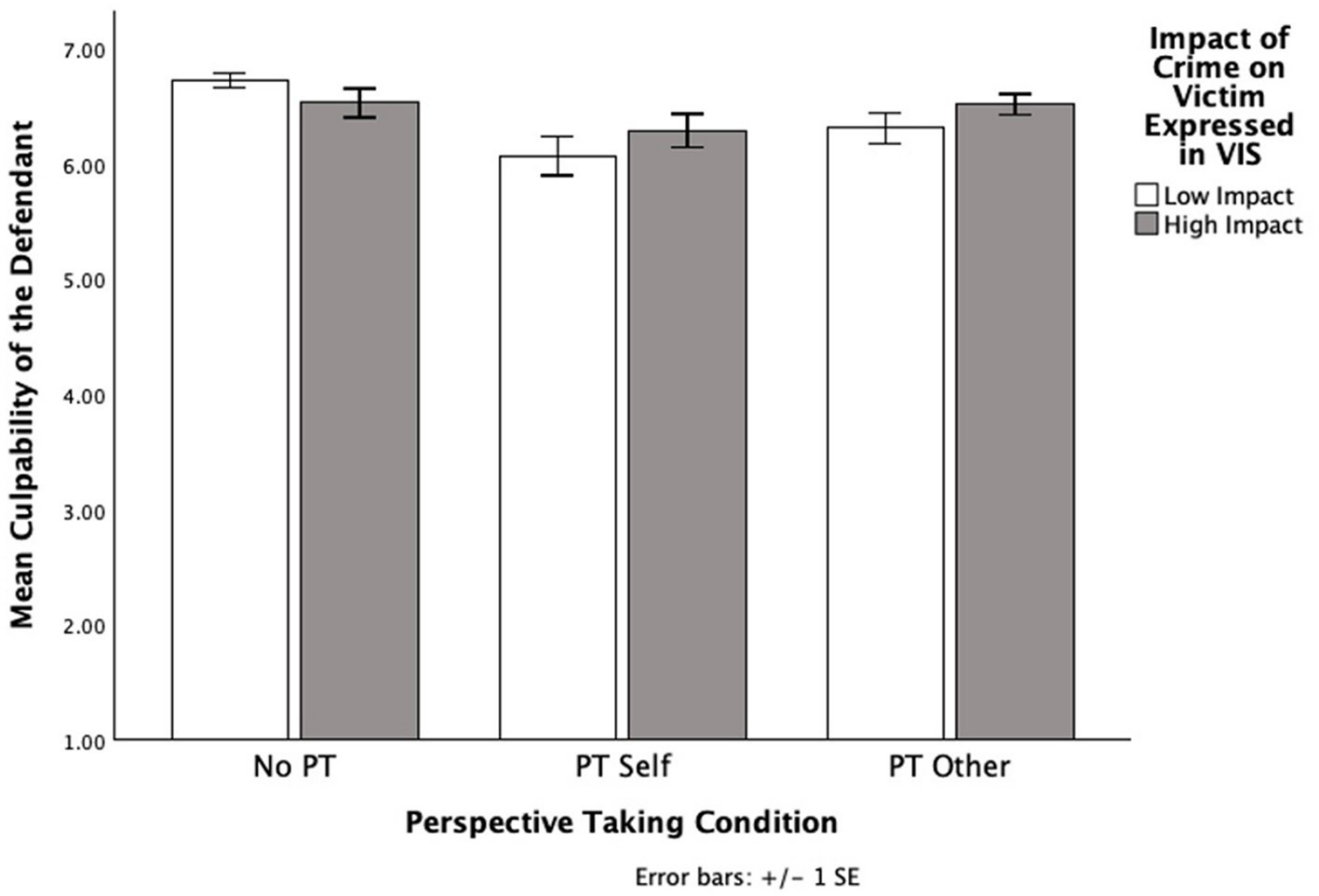
The effects of impact of crime on victim VIS and perspective taking on perceived culpability of a defendant in Experiment 3. PT means perspective taking. VIS means victim impact statement. There was a significant main effect for perspective taking, *p* = 0.010. PT self saw the defendant as less culpable than no PT, *p* = 0.013. No other pairwise comparisons for perspective taking were significant, *ps* < 0.184. There was no main effect for VIS (*p* = 0.500), nor was there a significant interaction between VIS and perspective taking (*p* = 0.315).

As in the previous experiments, there was also no significant interaction between VIS and perspective taking (*p* = 0.315). Unlike Experiments 1 and 2, there was also no main effect for VIS (*p* = 0.500) on culpability. However, there was a main effect for perspective taking on culpability, *F*_(1, 221)_ = 4.67, *p* = 0.010, $\eta_{\text{p}}^{2}$ = 0.041. As predicted and as seen in Figure 3, a Tukey's *post-hoc* analysis revealed that those who imagined the self (*M* = 6.20, *SD* = 1.01) saw the defendant as less culpable than non–perspective takers (*M* = 6.62, *SD* = 0.53), *t*_(221)_ = 2.75, *p* = 0.013, 95% CI (0.08, 0.77). No other pairwise comparisons were significant, *p*s > 0.184. For recidivism, there were no main effects for VIS (*p* = 0.919) or perspective taking (*p* = 0.576). There was also no interaction between perspective taking and VIS, *F*_(2, 226)_ = 2.92, *p* = 0.056, $\eta_{\text{p}}^{2}$ = 0.026.

### Empathy for defendant and victims

There were no effects of VIS, perspective taking, or any interaction between VIS and perspective taking on empathy for the defendant, *p*s > 0.332. For empathy with the victim, there were no effects of perspective taking and no interaction (*ps* > 0.268), but there was a main effect for VIS, *F*_(1, 221)_ = 4.31, *p* = 0.039, $\eta_{\text{p}}^{2}$ = 0.019, 95% CI (0.02, 0.77). Those who saw the high-impact VIS (*M* = 5.44, *SD* = 1.34) felt more empathy for the victim than those who saw the low-impact VIS (*M* = 5.10, *SD* = 1.40).

Consistent with Experiments 1 and 2, the VIS influenced empathy for the victim's family (in this case the fiancée), *F*_(1, 221)_ = 11.18, *p* < 0.001, $\eta_{\text{p}}^{2}$ = 0.048, 95% CI (0.29, 1.13). Those in the high-impact-VIS condition (*M* = 5.14, *SD* = 1.50) reported greater empathy than those in the low-impact-VIS condition (*M* = 4.46, *SD* = 1.57). There were no effects for perspective taking and no interaction between VIS and perspective taking, *p*s > 0.361.

### VIS emotionality and ability to perspective take

Confirming the manipulation, participants viewed the high-impact VIS (*M* = 5.27, *SD* = 1.39) as more emotional than the low-impact VIS (*M* = 4.25, *SD* = 1.60), *F*_(1, 220)_ = 27.20, *p* < 0.001, $\eta_{\text{p}}^{2}$ = 0.110, 95% CI (0.67, 1.49). Reported ease of taking the defendant's perspective was not influenced by either factor or their interaction (*p*s > 0.191). Similarly, the ability to perspective take with the victim was not influenced by either of the factors or their interaction (*p*s > 0.116). There was again an effect of VIS on the ability to perspective take with the victim's family, in this case the fiancée, *F*_(1, 220)_ = 5.20, *p* = 0.024, $\eta_{\text{p}}^{2}$ = 0.023, 95% CI (0.07, 1.00). Exposure to the high-impact VIS (*M* = 4.58, *SD* = 1.75) led to a greater ability to take the victim's fiancée's perspective than low-impact VIS (*M* = 4.05, *SD* = 1.63).

## Summary

Unlike Experiments 1 and 2, VIS impacted sentencing decisions but not culpability or recidivism. Participants also felt more empathy for the victim's family (as in Experiment 1) and the victim when the VIS expressed higher vs. lower impact. Perspective taking influenced perceptions of culpability such that when perspective takers imagined the self, then they saw the defendant as less culpable than non–perspective takers, but there was no difference between those who imagined the self and imagined the defendant. Because the trial used and the VIS manipulation were the same as in Experiment 1, there is no strong explanation for why there were effects for VIS on sentencing but not on culpability. The key difference between Experiments 1 and 3 was the perspective-taking manipulation; Experiment 3 manipulated the focus of perspective taking (self vs. other) when the target was the defendant whereas Experiment 1 manipulated the target of perspective taking. Speculatively, these findings suggest that the target of perspective taking and focus of perspective taking matter because, in Experiment 3, participants were only instructed to take the defendant's perspective by imagining the self or the other (the defendant). In Experiment 4, we expand this by examining a different perspective-taking manipulation that avoids explicit instructions.

## Experiment 4

In Experiments 1–3, perspective taking was manipulated by including prompts from defense attorneys or prosecutors (adapted from Skorinko et al., 2014). Instructing participants to perspective take is a common method used in past work more generally (e.g., Batson et al., 1997, 2002; Dovidio et al., 2004; Finlay and Stephan, 2000; Galinsky and Moskowitz, 2000; Gutierrez et al., 2014; Laurent and Myers, 2011; Myers et al., 2014; Shih et al., 2009, 2013; Skorinko and Sinclair, 2013; Stephan and Finlay, 1999; Todd et al., 2011; Vescio et al., 2003; Vorauer and Sasaki, 2014). Moreover, this method mimics how perspective taking might be introduced in a court case—with defense attorneys or prosecutors asking jurors to consider the perspectives of their clients (Minick, 2006).

However, factors either internal or external to the courtroom might also prompt jurors to spontaneously perspective take on their own, without any instructions. In the courtroom, the mindset to perspective take could be subtly cued in a variety of ways, such as through interactions with the judge, the attorneys, the presentation of the case, or the presentation of VISs (see Kirchmeier, 2008 Myers et al., 2018). Outside the courtroom, a question posed by an acquaintance (e.g., “What sort of person would do this?” or “Imagine how difficult this must be for them”) or a social media post might serve as primes for a perspective-taking mindset. Therefore, in Experiment 4, we adapt a mindset-priming technique to investigate how being in the mindset to perspective take with the defendant and how the impact of a crime's impact (i.e., in the VIS) influences perceptions of the defendant. We utilized a sentence-unscrambling task used in past work to manipulate perspective taking (Skorinko et al., 2012, 2023). We hypothesized that participants primed to take the defendant's perspective would perceive the defendant more favorably when presented with a low-impact VIS but more negatively when presented with a high-impact VIS. Based on the results of Experiments 1–3, we recognized that there might also be limited or no effects.

## Method

### Participants

A total of 170 individuals (49% female, 49% male, and 2% did not disclose) participated in this online experiment. All participants provided informed consent and were debriefed. Participants came from online (37.6% Amazon's Mechanical Turk and 47.6% SocialSci) and a university in the northeastern United States (14.7%). Those participating through an online platform were paid ($0.50 Mechanical Turk and $2 SocialSci) and those through the university earned course credit. Participants were randomly assigned participants to conditions with the goal of keeping cell sizes as even as possible. Among participants, 78% identified as White (9% Asian, 4% Black, 5% Latinx, 0.5% Middle Eastern, 0.5% Native American, 2% Multiracial or Other, and 1% did not disclose) and ranged in age from 17^16^ to 74 (*M* = 30.27, *SD* = 12.531). Participants were citizens of the United States (94%) and politically moderate (50%^17^), and about half were not engaged in higher education (52% not students, 47% university students, and 1% did not disclose). In total, 17 participants were removed from the analyses (8 were not U.S. citizens, 2 participants completed the study in < 1 min, one did not complete the unscrambling task, and six were likely bots based on open-ended responses). The analyses are based on 153 participants.

### Design and procedure

The experiment was a 2 (Perspective Taking: no perspective taking, perspective taking with defendant) × 2 (VIS: high vs. low impact) between participant design. We used the same murder trial and VISs used in Experiments 1 and 3. We used a simpler culpability measure by only measuring how responsible the defendant was for the crime.

### Procedure

After giving informed consent, participants were randomly assigned to unscramble 20 sentences that related to perspective taking with the defendant (e.g., “I understand the defendant's perspective”) or were neutral (“Toss the ball silently”; see Appendix E for the unscrambling tasks). Then participants were presented with the details of a murder trial and the VISs from the fiancée and the father that expressed a high or low impact of the crime on them. Participants then determined a sentence (life in prison or death penalty). In Experiment 4, we measured how responsible the defendant was for the crime on a 5-point Likert-type scale (1 = *Not at all*; 5 = *Very responsible*) rather than measuring culpability as in Experiments 1–3. Participants also indicated their empathy toward the defendant (α = 0.94), the victim (α = 0.93), and the victim's family (α = 0.97). We also measured the emotionality of the VISs (*r* = 0.37) and the ability to perspective take with the defendant (*r* = 0.51), the victim (*r* = 0.57), and the victim's family (α = 0.86). Participants provided demographic information including gender, citizenship, age, level of education, ethnicity, political orientation, and beliefs about the death penalty. Afterward, participants were thanked, debriefed, and awarded payment or credit.

## Results

Data were analyzed using 2 (VIS: high vs. low impact) × 2 (Perspective Taking: perspective taking vs. neutral prime) ANOVAs. Sentencing, which was a dichotomous variable, was examined using a generalized linear model that specified a binary logistic outcome.

### Sentencing

Participants were more likely to assign life in prison (*N* = 101) than the death penalty (*N* = 50), χ ^2^ (1, *N* = 151) = 17.23, *p* < 0.001. While neither perspective taking (*p* = 0.742) nor the interaction between VIS and perspective taking (*p* = 0.483) influenced sentencing, VIS did, χ ^2^ (1, *N* = 151) = 4.63 *p* = 0.033, ϕ = 0.17. More people in the high-impact-VIS condition believed a death sentence was appropriate (*n* = 31 vs. *n* = 19 in low-impact-VIS condition). This was reversed for life in prison sentences, with more people in the low-impact-VIS condition selecting life in prison (*n* = 57 vs. *n* = 44 in the high-impact-VIS condition).

### Responsibility of defendant

There were no main effects for VIS (*p* = 0.494), perspective taking (*p* = 0.855), and no interaction (*p* = 0.082).

### Empathy toward defendants and victims

There were no impacts of VIS, perspective taking or any interaction between VIS and perspective taking on empathy for the defendant, *p*s > 0.680. There were no main effects for perspective taking or VIS on empathy for the victim, *ps* > 0.414. Yet there was a significant interaction between VIS and perspective taking, *F*_(1, 149)_ = 4.12, *p* = 0.044, $\eta_{\text{p}}^{2}$ = 0.027. In the low-impact-VIS condition, perspective takers (*M* = 5.19, *SD* = 1.13) felt more empathy for the victim than non–perspective takers (*M* = 4.62, *SD* = 1.37), *F*_(1, 149)_ = 4.03, *p* = 0.046, $\eta_{\text{p}}^{2}$ = 0.026, 95% CI (0.01, 1.14). No other comparisons were significant, *ps* > 0.119. Unlike Experiments 1 and 3, there were no main effects or interaction on empathy for the victim's family, *ps* > 0.102.

### VIS emotionality and ability to perspective take

Participants again reported higher emotional content in the high-impact-VIS condition (*M* = 3.72, *SD* = 0.86) than the low-impact-VIS condition (*M* = 3.38, *SD* = 0.82), *F*_(1, 149)_ = 4.11, *p* = 0.013, $\eta_{\text{p}}^{2}$ = 0.040, 95% CI (0.07, 0.61). Neither perspective taking nor the interaction of perspective taking and VIS were significant, *p*s > 0.457. The ability to take the defendant's perspective was not influenced by either of the factors or their interaction, *p*s > 0.405. Likewise, neither factor nor their interaction had any effect on the ability to take the *victims'* perspectives, *ps*_*victim*_ >0.317 and *ps*_*victimfamily*_ > 0.158.

## Summary

Although more people believed that the defendant should receive life in prison rather than a death sentence, a greater number of people thought death was appropriate when exposed to a high-impact (vs. low-impact) VIS. However, the VIS did not influence perceptions of responsibility. In Experiment 4, we simplified the culpability measure to responsibility, and this may have contributed to the null effects. Although there was no effect for VIS on empathy toward the victim's family (contrary to Experiments 1 and 3), there was an interaction between VIS and perspective taking for empathy felt toward the victim. Perspective takers reported having more empathy for the victim than non–perspective takers, only in the low-impact-VIS condition. Self-reported perspective-taking ability was not influenced by the perspective-taking condition. The lack of effects for perspective taking may be due to the subtle nature of the prime manipulation. Likewise, the amount of time between the prime and the perspective-taking ability questions may have also influenced the self-reported perspective-taking ability.

## General discussion

Across four experiments, we investigated whether the impact of the crime expressed in a VIS and the perspective-taking target influenced perceptions of the defendants (see Table 1 for a summary of all results). The impact expressed in VISs tended to have more of an effect on jurors' perceptions than perspective taking. For instance, a guilty verdict was more likely to be assigned for high-impact (vs. low-impact) VISs (Experiment 2), and the death penalty tended to be given more when the impact was high rather than low (Experiments 3 and 4). In half of the experiments (Experiments 1 and 3), VISs also influenced perceptions of culpability with the defendant being perceived as more culpable in high-impact-VIS (compared to low-impact-VIS) conditions. Additionally, high-impact VISs elicited more empathy for (Experiment 3) and the ability to perspective take with the victim (Experiment 1) or the victim's family (Experiments 1 and 3) than low-impact VISs.

**Table 1:** Summary of crime used, perspective taking manipulation, VIS manipulation, and results in Experiments 1–4.

	**Experiment 1**	**Experiment 2**	**Experiment 3**	**Experiment 4**
Crime	Murder	Armed robbery	Murder	Murder
PT Manip	Def, vic, both, none	Def, vic, none	Imagine self, imagine other, none	• PT PRIME • Neutral prime
VIS Manip	High vs. low impact	High vs. low impact	High vs. low impact	High vs. low impact
Verdict	n/a	• Not guilty > guilt • VIS: high > guilty low	n/a	n/a
Sentence	Life > death	Null	• Life > death • VIS: high > death low	• Life > death • VIS: high > death low
Culpability	VIS: high > low	VIS: high > low	• Null • PT: imagine self < No PT	Null
Recidivism	Null	VIS: high > low	Null	n/a
**Empathy**				
Defendant	Null	VIS: low > high	Null	Null
Victim	Null	Null	VIS: high > low	PT ^*^ VIS: PT Low VIS > No PT Low VIS
Victim family	• VIS: high > low • PT: PT defendant lowest empathy	n/a	VIS: high > low	Null
Emotionality VIS	VIS: high > low	VIS: high > low	VIS: high > low	VIS: high > low
**PT Ability**				
Defendant	PT ^*^ VIS: high VIS, PT BOTH = greatest	VIS: low > high	Null	Null
Victim	VIS: high > low	Null	Null	Null
Victim family	• VIS: high > low • PT: PT defendant least ability to PT	n/a	VIS: high > low	Null

PT, perspective taking; VIS, victim impact statement; Manip, manipulation; Def, defendant; Vic, victim; n/a, main effects are designated by their abbreviations; VIS or PT, interaction is designated by PT ^*^ VIS.

Although effects were not consistent across all four experiments, this work is, to some extent, consistent with past VIS research (Greene et al., 1998; Luginbuhl and Burkhead, 1995; Matsuo and Itoh, 2016; Myers and Arbuthnot, 1999; Myers et al., 2002; Nuñez et al., 2017). For example, 29% of included experiments in a recent meta-analysis (Kunst et al., 2021) found effects of VIS on sentencing, but in the current work, VISs influenced death penalty sentencing in two thirds of the experiments that used a capital case. These findings also reaffirm arguments in past work that emotion and the impact of a crime on a victim can influence juror decision-making, thus warranting more attention in legal contexts (Bandes and Blumenthal, 2012; Blumenthal, 2005; Feigenson, 2016; Kunst et al., 2021; Myers and Greene, 2004; Nuñez et al., 2015, 2016; Salerno and Bottoms, 2009; Salerno, 2021).

Contrary to our hypotheses, the effects of perspective taking were limited. Perspective taking only influenced culpability in Experiment 3, where those in the perspective-taking-self condition viewed the defendant as less culpable than non–perspective takers. These findings indicate that when VISs are presented, perspective taking is not likely to influence verdicts and sentencing (including death penalty decisions). But, if perspective takers imagine themselves in the shoes of the defendant, they are likely to perceive the defendant as less culpable than non–perspective takers, potentially because this type of perspective taking does not cue self-evaluation (Vorauer and Sasaki, 2014). Yet there was no difference between the perspective-taking-self and perspective-taking-other conditions. Therefore, future research should further examine ways to manipulate the type of perspective taking and its effects on juror decision-making.

Unlike past work (Skorinko et al., 2014), perspective takers did not report greater empathy for the defendant, nor did empathy mediate downstream outcomes. One difference between the current and past work is the presentation of VISs, which may impact empathy felt toward the defendant, as suggested in past research (Kirchmeier, 2008; Myers et al., 2018). Another difference is the type of crime, as past work on perspective taking used vehicular manslaughter and hit-and-run cases (Skorinko et al., 2014), but the current work used a capital murder case in which the defendant was found guilty and a non-capital armed robbery case. Future research should more directly investigate whether VISs contribute to empathy felt toward the defendant when perspective taking with the defendant and whether the type of crime matters.

The perspective-taking manipulation did not always result in a greater self-reported ability to perspective take with defendants. In Experiments 3 and 4, asking participants to take the defendant's perspective did influence participants' ability to take the defendant's perspective. Yet when exposed to high-impact VISs in Experiment 1 and asked to perspective take with both the defendant and the victims, participants reported a greater ability (relative to all other conditions, which did not differ) to take the defendant's perspective. Although the perspective-taking manipulation did not always increase the self-reported ability to do so, this is consistent with past work that found that perspective takers are not more likely to report perspective taking than those in control conditions (Ferguson, 2008; Skorinko et al., 2023). In the current studies, there may have been too much time between when perspective taking was induced (i.e., beginning of the experiments) and when it was measured (i.e., at the end), thereby affecting reports of experiencing perspective taking (Hauser et al., 2018). Future research should consider moving the perspective-taking manipulation check earlier. Speculatively, another reason for the null effects of perspective-taking instructions is that the trial used in the current work was longer than in previous research (Skorinko et al., 2014) and may have obscured any effects. Future research should consider using multiple prompts or finding other ways to strengthen perspective-taking manipulations when study materials are longer. Likewise, how effective the perspective-taking prime used in Experiment 4 was is unclear. Future research should consider other priming techniques, such as a mindset-priming procedure (Chen et al., 1996).

## Other limitations and future research

A limitation of the current work is that it did not investigate additional factors that might influence perspective taking and VISs, such as race/ethnicity, gender, respectability, and SES of defendants and victims. In the current work, the defendant and victim were always male, and victim SES was unclear (a mini-mart cashier but in medical school). Understanding the effects of target factors is important because in real trials jurors can see defendants (and sometimes victims), likely making these factors more salient. In addition, past research shows that respectability, SES, race, gender, racial stereotypes about who is a victim, racial identity, and the racial composition of juries can influence juror perceptions and decisions (Forsterlee et al., 2011; Gordon et al., 1996; Greene et al., 1998; Hymes et al., 1993; Johnson et al., 2020; Schweitzer and Nuñez, 2017; Skorinko and Spellman, 2013; Sommers, 2006; Sunnafrank and Fontes, 1983; Sweeney and Haney, 1992; Williams and Holcomb, 2001; Willis Esqueda, 1997). Investigating these factors is also important because past work shows that perspective taking sometimes leads to prejudice reductions, but not always (see Paluck et al., 2021, for a review), and prejudice is likely an important factor in the decisions jurors make regarding defendants. Thus, factors such as these would plausibly moderately the impacts of perspective-taking and VIS manipulations on juror decision-making.

While the current work looked at the impact of the crime on the victim, it did not consider the injury severity as most experiments used a capital murder case. Because injury severity influences juror decision-making (Vallano and McQuiston, 2018) and likely signals the impact of the crime on the victim, future research should investigate this factor. Future research may also examine additional participant characteristics, such as participants' moral values and moral emotions (such as anger or sadness; Laurent et al., 2016; Niemi and Young, 2016; Salerno, 2021). Moral values and emotions should plausibly influence how participants respond to VIS and perspective-taking instructions and consequently might influence blame placed on the defendant or victim (Niemi and Young, 2016; Salerno, 2021). Beyond this, most research on VISs has used a written form; therefore, future research should examine whether format matters (as suggested by Kunst et al. (2021). This is important because audio and visual formats introduce additional factors, such as how the victim speaks (e.g., accent), looks, and behaves, and could also allow for other ways to manipulate perspective taking.

The current work was conducted in the United States, a country with a common-law legal system (i.e., an adversarial system that distinguishes between guilt and sentencing phases; Kunst et al., 2021), and little research has been conducted in countries that use a civil-law system (i.e., an inquisitive system that does not distinguish guilt and sentencing phases). Thus, as recommended by Kunst et al. (2021), future research needs to extend beyond the United States and even common-law legal systems and investigate the role of VISs in countries with a civil-law system (e.g., South Korea and Austria).

Also, the samples used here were a mix of online community members and university students who were jury-eligible (U.S. citizens over 18 years of age). We recognize that this may not reflect the composition of real juries. It has also been argued that low power from smaller sample sizes may be an issue in detecting the effect of perspective taking (in relation to prejudice reduction; Huang et al., 2021). In the current work, we ran as many participants as possible the given logistics and resources available (see Laken, 2022). Moreover, the conclusions are based on multiple experiments, and we report effect sizes that might help guide power considerations for future researchers. However, we acknowledge that larger samples of actual potential jurors (e.g., people who have been called to jury duty but are dismissed without serving) would be useful for drawing firmer conclusions from our own work and others' work. Also, Experiment 2 used a different trial than Experiments 1, 3, and 4. The differences in empathy felt for the defendant that emerged in Experiment 2 and the differences in reported perspective-taking ability are possibly vignette-specific. Although developing trial materials that are similar for capital and non-capital cases may be difficult, at least attempting to rise to the challenge is worthwhile.

In conclusion, the current work provides additional evidence that the impacts of a crime on victims expressed in VISs have some influence on mock jurors' perceptions and decisions for defendants in capital and non-capital cases. Yet contrary to our predictions and past work, perspective taking had only limited effects on jurors' perceptions across impact levels of VISs. Overall, this work reasserts the need for the courts to understand the roles and impacts that content in VIS content and emotional content more generally can have on jurors and trial proceedings (Bandes and Blumenthal, 2012; Blumenthal, 2005; Feigenson, 2016; Myers and Greene, 2004; Nuñez et al., 2015, 2016; Salerno and Bottoms, 2009; Salerno, 2021).

## Data Availability

The datasets presented in this study can be found in online repositories. The names of the repository/repositories and accession number(s) can be found below: https://osf.io/wkyqn/.

## Footnotes

^1^*Booth v. Maryland*, 482 U.S. 496 (1987).

^2^South Carolina v. Gathers, 490 U.S. 805 (1989).

^3^*Payne v. Tennessee*, 501 U.S. 808 (1991).

^4^Participants were recruited from multiple platforms because one platform stopped offering its services during data collection, forcing a change in recruitment procedures. Sample sizes are not large enough to make sample comparisons, but no substantive differences emerged in any experiment when analyses accounted for differences in sample composition.

^5^The three participants who were 17 years old were university students with parental permission to participate.

^6^Political orientation was measured on a 7-point scale; those who indicated a “1” or “2” were classified as conservative; those indicating a “3”, “4”, or “5” were classified as moderate; and those indicating a “6” or “7” were classified as liberal. The item was not assessed in Experiment 3.

^7^Along with demographics, we included 14 questions from the Juror Bias Scale (Kassin and Wrightsman, 1983). As this was not central to the presented work, details about this measure are included in Appendix C.

^8^If all participants were included in the analyses, there were no substantive differences in the results in any of the four experiments.

^9^Due to a coding error, the “how moved” item was not administered for the victim's fiancée or father.

^10^Except for a slight difference in terminology, this approach is the same as using a logistic regression where all categorical predictors and their interactions have been effects coded. The same conclusions were reached in all studies when using logistic regression.

^11^We examined empathy as a mediator (as in Skorinko et al., 2014). Empathy was not a significant mediator in any of the four experiments. Therefore, the role of empathy as a mediator is not discussed further.

^12^In addition, 15% were politically conservative, and 36% were moderate.

^13^The three participants who were 17 had parental permission to participate in the study.

^14^As in Experiment 1, empathy with the fiancée did not include the “how moved” item. Empathy toward the victim's father was not measured in Experiment 3.

^15^Due to a coding error, participants only answered “how easily could you put yourself in the defendant's shoes”, as the motivation to perspective take item was erroneously not available.

^16^The one participant who was 17 had parental consent to participate.

^17^In addition, 28% were politically conservative, and 21% were liberal.
