# Supplementary material for: Victim impact statements limit the effects of perspective taking on juror decisions
Source: Front Cognit. 2024 Nov 20;3:1430999. doi: 10.3389/fcogn.2024.1430999 (PMC13281215; doi:10.3389/fcogn.2024.1430999)
Supplement: Supplementary file 1 [file Data_Sheet_1.docx]

Running Head: PERSPECTIVE TAKING AND VICTIM IMPACT STATEMENTS

Supplemental Materials for

Victim Impact Statements Limit the Effects of Perspective Taking on Juror Decisions

Jeanine L. M. Skorinko^1^*, Sean Laurent^2^, Emily Bendremer^1+^, Kaitlyn Schneider^1+^, Valicia Hale^1^, Alisionna Iannacchione^1^, Julia LaValley^1^,

Gizem Dermircioglu^1^, Melissa Paris^1^, Kyla Wesley^1^, Angelica DeFreitas^1^, & Jeremy Blumenthal^3^

Worcester Polytechnic Institute^1^, The Pennsylvania State University^2^ , & Syracuse University College of Law^3^,

**Appendix A**

**Perspective Taking Prompts in Experiment 1**

**No Perspective Taking Condition:**

“The defense has offered evidence for you to consider when deciding the penalty for George Taylor. All of the materials the defense attorney is presenting are considered "mitigating circumstances." A mitigator is a reason to consider life in prison without the possibility of parole as opposed to the death penalty.”

**Perspective Taking with the Defendant Condition:**

“The defense has offered evidence for you to consider when deciding the penalty for George Taylor. The defense also asks you to try to take the defendant, George Taylor's perspective, imagining how he feels to be in this situation, especially when considering the mitigating circumstances involved in this case. All of the materials the defense attorney is presenting are considered "mitigating circumstances." A mitigator is a reason to consider life in prison without the possibility of parole as opposed to the death penalty.”

**Perspective Taking with the Victim Condition:**

“The prosecution has offered evidence for you to consider when deciding the penalty for George Taylor. All of the issues the prosecutor presents are official "aggravating circumstances" in New Jersey. An aggravator is a legal reason to favor the death penalty over the sentence of life in prison without the possibility of parole. The prosecution also asks you to take the victim, Matthew Dunning’s perspective, imagining how he feels to be in this situation, especially when considering the aggravating circumstances involved in this case.”

**Perspective taking with Both Defendant and Victim Condition:**

*Note: Participants were given the prompt to perspective with the victim as well as the prompt to perspective take with the defendant. Thus, participants read the closing arguments for the prosecution and the perspective taking prompt and then the closing arguments for the defense and the perspective taking prompts. The perspective taking prompts were the same as in the perspective taking victim and perspective taking defendant conditions.*

***Perspective Taking with Victim Prompt***

“The prosecution has offered evidence for you to consider when deciding the penalty for George Taylor. All of the issues the prosecutor presents are official "aggravating circumstances" in New Jersey. An aggravator is a legal reason to favor the death penalty over the sentence of life in prison without the possibility of parole. The prosecution also asks you to take the victim, Matthew Dunning’s perspective, imagining how he feels to be in this situation, especially when considering the aggravating circumstances involved in this case.”

***Perspective Taking with the Defendant Prompt:***

“The defense has offered evidence for you to consider when deciding the penalty for George Taylor. The defense also asks you to try to take the defendant, George Taylor's perspective, imagining how he feels to be in this situation, especially when considering the mitigating circumstances involved in this case. All of the materials the defense attorney is presenting are considered "mitigating circumstances." A mitigator is a reason to consider life in prison without the possibility of parole as opposed to the death penalty.”

**Appendix B**

**Victim Impact Statements in Experiments 1, 3, and 4**

**Sample Low Impact Victim Impact Statement**

MS. BENNETT [Prosecutor]: The State calls Jennifer Thomas.

JENNIFER THOMAS, having been first duly sworn, testified as follows:

DIRECT EXAMINATION BY MS. BENNETT:

Q. You're the same Jennifer Thomas that has also testified in this trial?

A. I am.

Q. Jennifer, when did you find out that Matthew was dead?

A. I found out on Monday, September 18th, while I was at work.

Q. How did you find out?

A. I received a page, which later I realized turned out to be from the detective and someone from a secretary at work and I called back, was told on the phone by the Department Head that Matthew had been found dead.

Q. Did you and Matthew have plans to become engaged?

A. Yes, we did. We had planned on it early on in our relationship. The first six months it was just the matter of logistics ending up in the same circumstances, which we finally had. We planned to be married by the end of his first year of medical school, and engaged, I am not sure when.

Q. Thank you, Jennifer.

THE COURT: You may step down, Miss Thomas.

*******************

MS. BENNETT: The State calls Doctor Gregory Dunning.

GREGORY DUNNING, having been first duly sworn, testified as follows:

DIRECT EXAMINATION BY MS. BENNETT:

Q. Could you please state your full name for the jury?

A. I’m Gregory S. Dunning.

Q. And how are you employed?

A. I'm a physician, a neurologist with the University Health Care System. I'm also an associate professor at the University Medical School.

Q. You live in Hampshire, correct?

A. Hampshire, yes.

Q. And who do you live there with?

A. I live with Caroline Dunning, my wife.

Q. Does your wife work?

A. Caroline worked full time very productively prior to Matthew's death. She’s a licensed social worker with an MSW. With Matthew's death, she has not been able to work full time. She's had a couple of brief part-time jobs, also, in social work.

Q. And did you go to work on September 18, 2000?

A. Yes, I did.

Q. And how was it, sir, that you found out about the death of your youngest son?

A. The police called my office. I was in the clinic, though, so my secretary then passed the word along that there was a problem with Matthew . . . and I was told to return to my office. I went back up there. There was nobody there, so at that point I called. I called Caroline to find out what the problem was and, at that point, she told me over the phone that Matthew had been shot.

Q. Doctor Dunning, can you tell the members of the jury what the impact of this crime has been on you?

A. Devastating. I lost a considerable amount of work. I have had difficulty keeping up subsequent to that. I don’t know if I answered your question.

Q. You did. You did. Thank you, Doctor Dunning.

THE COURT: You may step down, sir.

MS. BENNETT: Your Honor, at this time the State rests.

**Sample High Impact Victim Impact Statement**

MS. BENNETT [Prosecutor]: The State calls Jennifer Thomas.

JENNIFER THOMAS, having been first duly sworn, testified as follows:

DIRECT EXAMINATION BY MS. BENNETT:

Q. You're the same Jennifer Thomas that has also testified in this trial?

A. I am.

Q. Jennifer, when did you find out that Matthew was dead?

A. I found out on Monday, September 18th, while I was at work.

Q. How did you find out?

A. I received a page, which later I realized turned out to be from the detective and someone from a secretary at work and I called back, was told on the phone by the Department Head that Matthew had been found dead.

Q. Did you and Matthew have plans to become engaged?

A. Yes, we did. We had planned on it early on in our relationship. The first six months it was just the matter of logistics ending up in the same circumstances, which we finally had. We planned to be married by the end of his first year of medical school, and engaged, I am not sure when.

Q. Would you tell the members of the jury what the impact of this crime has had on you?

A. There are obviously immediate impacts and also long term impacts. I lost my best friend. Changed my life on a daily basis. You know, jeopardized my position at work, took time off, received counseling . . . but even more so it really destroyed the future that Matt and I had planned for each other, to be together. So I lost, you know, my husband that I would have spent the rest of my life with and the family that we had dreamed about. We already discussed how we wanted to raise our family, where we'd live so we'd be close to his family, close to my family. They were definite plans that were interrupted and dreams that were destroyed.

Q. Thank you, Jennifer.

THE COURT: You may step down, Miss Thomas.

*******************

MS. BENNETT: The State calls Doctor Gregory Dunning.

GREGORY DUNNING, having been first duly sworn, testified as follows:

DIRECT EXAMINATION BY MS. BENNETT:

Q. Could you please state your full name for the jury?

A. I’m Gregory S. Dunning.

Q. And how are you employed?

A. I'm a physician, a neurologist with the University Health Care System. I'm also an associate professor at the University Medical School.

Q. You live in Hampshire, correct?

A. Hampshire, yes.

Q. And who do you live there with?

A. I live with Caroline Dunning, my wife.

Q. Does your wife work?

A. Caroline worked full time very productively prior to Matthew's death. She’s a licensed social worker with an MSW. With Matthew's death, she has not been able to work full time. She's had a couple of brief part-time jobs, also, in social work but has really been markedly disabled with depression as well.

Q. And did you go to work on September 18, 2000?

A. Yes, I did.

Q. And how was it, sir,that you found out about the death of your youngest son?

A. The police called my office. I was in the clinic, though, so my secretary then passed the word along that there was a problem with Matthew . . . and I was told to return to my office. I went back up there. There was nobody there, so at that point I called. I called Caroline to find out what the problem was and, at that point, she was barely able to tell me over the phone that Matthew had been shot.

Q. Doctor Dunning, can you tell the members of the jury what the impact of this crime has been on you?

A. Devastating. . . . I lost a considerable amount of work. I have had difficulty keeping up subsequent to that. My wife has been disabled not only from the depression and grief but the few months later that she developed an attack of multiple sclerosis; we had to arrange for treatment with her at the hospital and . . . I know that my oldest son also was just very upset. We had numerous conversations. I don’t know if I answered your question.

Q. You did. You did. And the impact that it has had on you, the impact that it has had on your wife, how has that affected your family life?

A. There's almost no words to describe the pain. It's hard to lose a child. For a child to precede you in death is the most horrible thing I can think to happen to a parent. And for it to happen in this way, for him to be so brutally murdered and to know the horror he went through before he died is - I can never - I'll never get over it. Never. You know, you raise a child up to be right with God, right with your family and right with their community, and Matthew was a shining example of all of that. We were so proud of him and so proud of all that he had done and all that he had accomplished and what kind of person he was. We came to rely on him for the kind of a person that he was, though he was just a boy, just a young boy, and to have that hole and have that not there . . . .Our lives will never be the same. There's emptiness that nothing or no one can fill.

Q. Thank you, Doctor Dunning.

THE COURT: You may step down, sir.

MS. BENNETT: Your Honor, at this time the State rests.

**Appendix C**

**Exploratory Items Used in Experiments 1-4**

***Perceptions of Victim, Self-Other Overlap, Interpersonal Reactivity Index, and Juror Bias Scale (Experiments 1, 2, 3, & 4)***

In all experiments, we included a few additional measures that have been relevant in the perspective taking literature or VIS literature. These measures were not part of our central hypotheses, but we included them for exploratory purposes. Since past research (Skorinko et al., 2014) measured both perceptions of the defendant and of the victim, we wanted to be consistent with this past work; therefore, in addition to perceptions of the defendant reported in the manuscript, we also measured perceptions of the victim by having participants indicate how responsible, blameworthy, and at fault the victim was for the crime on 7-point Likert-Type scales from 1 (Not at all) to 7 (Extremely). These items were averaged together indicating more negative perceptions of the victim. This was in all four experiments.

One factor that has received a lot of attention in the perspective taking literature is self-other overlap (Davis et al., 1996; Galinsky et al., 2005; Galinsky et al., 2008; Galinsky & Moskowitz, 2000; Goldstein & Cialdini, 2007; Hodges et al., 2011; Laurent & Myers, 2011; Skorinko et al., 2012). Self-other overlap occurs when perspective takers see themselves as more similar to targets, whether that is seeing the target as more “self-like” or seeing oneself as more “target-like”. This perceived overlap can be a driving force in changing perspective takers’ beliefs about themselves and others (Laurent & Myers, 2011; Myers et al., 2014). However, this has not been examined in the legal setting. Therefore, we wondered if self-other overlap was experienced with the defendant and the victim and if that played a role in perceptions of the defendant. To measure this, participants viewed seven depictions of circles that overlapped to varying degrees and chose the representation that best fit how they saw themselves and the defendant (and the victim; adapted from Aron et al., 1992). Participants also indicated on a 7-point Likert-Type(1 = Not at All; 7 = Very Much) how similar they believed they were with the defendant (also the victim). We averaged the responses to the circle measure and the similarity question to create a composite for self-other overlap for the defendant (and the victim) with higher numbers indicating more self-defendant or self-victim overlap. We did not include this measure in Experiment 4.

Another question that comes up in the perspective taking literature is whether individual differences in perspective taking ability play a role. Therefore, we utilized the Interpersonal Reactivity Scale (Davis, 1980) to measure individual differences in perspective taking and empathic concern (see below for the items) and to see if this had any influence on the results. Items were reverse scored as needed and then all items were averaged together to create a score for Perspective Taking and a separate score for Empathic Concern. Higher numbers indicate a tendency to perspective take or have empathic concern. This was measured in all four experiments.

Finally, since the experiments had participants read trials (often times a murder trial where the defendant was found guilty), we also wanted to examine if any pre-existing biases towards the defense or prosecution influenced perceptions of the defendant. Therefore, we included 14 questions from the Juror Bias Scale (Kassin & Wrightsman, 1983) to examine any biases participants might have towards the defense or the prosecution (7-point Likert-Type Scale; 1 = Strongly Disagree; 7 = Strongly Agree). Six questions measured bias for the defense and eight questions measured a preference for the prosecution (see below for items). All eight pro-prosecution items were reverse scored. All items were then averaged such that higher numbers indicated more bias towards the defense. In Experiment 4, given the limited effects in the previous experiments, we did not measure self-other overlap or juror bias. We did include the 14 items from the Interpersonal Reactivity Scale (Davis, 1980). This was not included in Experiment 4.

***Crime Severity, Need for Affect, Need for Cognition (Experiment 2)***

Based on past research in legal decision making and VIS, we included additional exploratory measures in Experiment 2. One factor that appears to influence juror decision making is the severity of the crime, which includes physical and psychological injuries (Vallano et al., 2012; Vallano & McQuiston, 2018; Vallano & Slapinski, 2018). Since, in Experiment 2, the crime did not involve murder but rather armed robbery, we wanted to be able to see if perceptions of the severity of the crime mattered. Therefore, participants considered the severity of the crime, the physical injuries, the psychological injuries, and the financial strain on the victim on a 7-point Likert-Type scale (1 = Not At All; 7 = Very Severe).

Wevodau, Cramer, Clark, and Kehn (2014) found that participants with a higher need for affect gave higher sentencing recommendations than those with lower need for affect when VIS was delivered; however, the need for cognition did not influence sentencing decisions. Given these findings, we also wanted to see if the Need for Affect (Maio & Esses, 2001) or the Need for Cognition (Cacioppo, & Petty, 1982) influenced the results, especially since the crime being used was not murder but rather armed robbery (not a common crime used in the VIS literature). Participants also completed 10 items from the Need for Affect Scale (Maio & Esses, 2001) with five questions measuring approaching emotions and five questions measuring avoidance of emotions. We also used four items from the Need for Cognition Scale (Cacioppo, & Petty, 1982) to measure an individual’s desire (or need) to think or engage in cognitive activities.

**References:**

Aron, A., Aron, E. N., & Smollan, D. (1992). Inclusion of other in the self scale and the structure of interpersonal closeness. Journal of Personality and Social Psychology, 63(4), 596–612. [https://doi.org/10.1037/0022-3514.63.4.596](https://psycnet.apa.org/doi/10.1037/0022-3514.63.4.596)

Cacioppo, J. T., & Petty, R. E. (1982). The need for cognition*. Journal of Personality and Social Psychology, 42* (11), 116-131. <https://doi.org/10.1037/0022-3514.42.1.116>

Davis, M. H. (1980). Interpersonal Reactivity Index (IRI) [Database record]. APA PsycTests. <https://doi.org/10.1037/t01093-000>

Davis, M. H., Conklin, L., Smith, A., & Luce, C. (1996). Effect of perspective taking on the cognitive representation of persons: A merging of self and other. Journal of Personality and Social Psychology, 70(4), 713–726. <https://doi.org/10.1037/0022-3514.70.4.713>

Galinsky, A. D., Ku, G., & Wang, C. S. (2005). Perspective-taking and self-other overlap: Fostering social bonds and facilitating social coordination. *Group Processes & Intergroup Relations*, *8*(2), 109-124. <https://doi.org/10.1177/1368430205051060\>

Galinsky, A. D., & Moskowitz, G. B. (2000). Perspective-taking: Decreasing stereotype expression, stereotype accessibility, and in-group favoritism. *Journal of Personality and Social Psychology*, *78*(4), 708-724. <https://doi.org/10.1037/0022-3514.78.4.708>

Galinsky, A. D., Wang, C. S., & Ku, G. (2008). Perspective-takers behave more stereotypically. *Journal of Personality and Social Psychology,* *95*(2), 404-419. <https://doi.org/10.1037/0022-3514.95.2.404>

Goldstein, N. J., & Cialdini, R. B. (2007). The spyglass self: A model of vicarious self-perception. *Journal of Personality and* *Social Psychology, 92,* 402-417. <https://doi.org/10.1037/0022-3514.92.3.402>

Hodges, S.D., Clark, B.A., Myers, M.W. (2011). Better living through perspective taking. In: Biswas-Diener, R. (Eds) *Positive Psychology as Social Change*. Springer, Dordrecht. <https://doi.org/10.1007/978-90-481-9938-9_12>

Laurent, S. M. & Myers, M. W. (2011). I know you’re me, but who am I? Perspective taking and seeing the other in the self. *Journal of Experimental Social Psychology, 47*, 1316-1319. <https://doi.org/10.1016/j.jesp.2011.05.018>

Myers, M. W., Laurent, S. M., & Hodges, S. D. (2014). Perspective taking instructions and self-other overlap: Different motives for helping. Motivation and Emotion, 38(2), 224–234. [https://doi.org/10.1007/s11031-013-9377-y](https://psycnet.apa.org/doi/10.1007/s11031-013-9377-y)

Skorinko, J. L., Sinclair, S., & Conklin, L. (2012). Perspective taking shapes the impact of significant-other representations. *Self and Identity, 11*(2), 170-184. <https://doi.org/10.1080/15298868.2010.517986>

Vallano, J. P., Winter, R. J., & Charman, S. D. (2013). Is this injury reasonable? Do psychological injury expectations affect mock jurors’ legal decisions in a sexual harassment case? *Psychiatry, Psychology and Law, 20*(6), 834-852. <https://doi.org/10.1080/13218719.2012.744626>

Vallano, J. P., & McQuiston, D. E. (2018). An exploration of psychological and physical injury schemas in civil cases. *Applied cognitive psychology*, *32*(2), 241-252. <https://doi.org/10.1002/acp.3399>

Vallano, J. P., & Slapinski, K. A. (2018). The impact of psychological injury evidence and jurors’ schemas on civil case decisions. *Psychological Injury and Law*, *11*(4), 362-369. https://doi.org/10.1007/s12207-018-9330-1

Wevodau, A. L., Cramer, R. J., Clark, J. W., & Kehn, A. (2014). The role of emotion and cognition in juror perceptions of victim impact statements. *Social Justice Research, 27* (1), 45–66. https://doi.org/10.1007/s11211-014-0203-9

**Interpersonal Reactivity Index (IRI) Items**

Davis (1980)

1. I often have tender, concerned feelings for people less fortunate than me. (EC)
2. I sometimes find it more difficult to see things from the “other guys” view. (PT_Reverse)
3. Sometimes I don’t feel very sorry for other people when they are having problems. (EC_Reverse)
4. I try to see everybody’s side of a disagreement before I make a decision. (PT)
5. When I see someone being taken advantage of, I feel kind of protective towards them. (EC)
6. I sometimes try to understand my friends better by imaging how things look from their perspective. (PT)
7. Other people’s misfortunes do not usually disturb me a great deal. (EC_Reverse)
8. If I’m sure I’m right about something, I don’t waste time listening to other people’s arguments. (PT_Reverse)
9. When I see someone being treated unfairly, I sometimes don’t feel much pity for them. (EC_Reverse)
10. I believe that there are two sides to every question and I try to look at them both. (PT)
11. When I’m upset with someone, I try to “put myself in their shoes” for a while. (PT)
12. I am often quite touched by things I see happen. (EC)
13. I would describe myself as a pretty softhearted person. (EC)
14. Before criticizing someone, I try to imagine how I would feel if I were in their place. (PT)

Note:

EC = Empathic Concern

PT = Perspective Taking

**Juror Bias Scale**

Kassin & Wrightsman (1983)

1. Too often jurors hesitate to convict someone who is guilty out of pure sympathy. (P)
2. The death penalty is cruel and inhumane. (D)
3. For serious crimes like murder, a defendant should be found guilty if there is a 90% chance that he has committed a crime. (P)
4. Defense lawyers don’t really care about guilty or innocence, they are just in the business to make money. (P)
5. The defendant is often a victim of his own bad reputation. (D)
6. If a grand jury recommends that a person be brought to trail, then that person probably committed the crime. (P)
7. Extenuating circumstances should not be considered – if a person commits a crime, than that person should be punished. (P)
8. A prior record of conviction is the best indicator of a person’s guilt. (P)
9. Too many innocent people are wrongfully imprisoned. (D)
10. Once a criminal, always a criminal. (P)
11. Circumstantial evidence is too weak to use in court. (D)
12. If a majority of the evidence – but not all of it – suggests that the defendant committed the crime, the jury should vote not guilty. (P)
13. A defendant should be found guilty if 11 out of 12 jurors vote guilty. (P)
14. In most cases where the accused presents a strong defense, it is only because of a good lawyer. (P)

Note:

P = Prosecution Bias

D = Defense Bias

**Need for Affect Scale**

Maio & Esses (2001)

1. If I reflect on my past, I see that I tend to be afraid of feeling emotions. (AVOID)
2. I feel that I need to experience strong emotions regularly. (APPROACH)
3. Emotions help people to get along in life. (APPROACH)
4. I find strong emotions overwhelming and therefore try to avoid them. (AVOID)
5. I think that it is important to explore my feelings. (APPROACH)
6. I would prefer not to experience either the lows or highs of emotion. (AVOID)
7. I do not know how to handle my emotions, so I avoid them. (AVOID)
8. It is important for me to be in touch with my feelings. (APPROACH)
9. It is important for me to know how others are feeling. (APPROACH)
10. Emotions are dangerous—they tend to get me into situations I would rather avoid. (AVOID).

**Need for Cognition Scale**

Cacioppo, & Petty (1982)

1. I would prefer complex to simple problems.
2. I like to have the responsibility of handling a situation that requires a lot of thinking.
3. Thinking is not my idea of fun. (Reverse Score)
4. I would rather do something that requires little thought than something that is sure to challenge my thinking abilities. (Reverse Score)
5. I try to anticipate and avoid situations where there is likely a chance I will have to think in depth about something. (Reverse Score)
6. I find satisfaction in deliberating hard and for long hours.
7. I only think as hard as I have to. (Reverse Score).
8. I prefer to think about small, daily projects to long-term ones. (Reverse Score)
9. I like tasks that require little thought once I've learned them. (Reverse Score)
10. The idea of relying on thought to make my way to the top appeals to me.
11. I really enjoy a task that involves coming up with new solutions to problems.
12. Learning new ways to think doesn't excite me very much. (Reverse Score)
13. I prefer my life to be filled with puzzles that I must solve.
14. The notion of thinking abstractly is appealing to me.
15. I would prefer a task that is intellectual, difficult, and important to one that is important but does not require much thought.
16. I feel relief rather than satisfaction after completing a task that requires a lot of mental effort. (Reverse Score)
17. It's enough for me that something gets the job done; I don't care how or why it works. (Reverse Score)
18. I usually end up deliberating about issues even when they do not affect me personally.

**Appendix D**

**Victim Impact Statements for Armed Robbery Case in Experiment 2**

**Low Impact VIS**

The Prosecution called the additional witness:

MS. BENNETT [Prosecutor]: The State calls Matthew Dunning.

MATTHEW DUNNING, having been first duly sworn, testified as follows:

DIRECT EXAMINATION BY MS. BENNETT:

Q. Mr. Dunning, could you please tell the court what happened on September 18, 2019?

A. Yes. On September 18, 2019, Mr. Taylor entered the Quick Stop gas station where I was working as a cashier. Mr. Taylor pulled a gun on me and yelled at me to open the register and put the money on the counter. I did what he wanted. I asked him to stay calm, but he kept yelling. Then, he told me to get back and after I did, he shot me.

Q. And what did Mr. Taylor do after that?

A. Umm, well, I heard him grab the cash and run out the door.

Q. You “heard” him. You didn’t see him? What were you doing at that point?

A. Well, I was lying on the floor.

Q. Mr. Dunning, could you describe to us the impact this crime has had on you?

A. Things have been difficult. I hate quiet now. I have to leave the TV on to sleep and I still jump at every loud noise. I was also diagnosed with post-traumatic stress disorder a few months after I was shot. I have been in counseling since, but I still have not been able to return to work.

Q. Have you been able to return to school? You attend Rutgers Medical School, is that correct?

A. Yes, that is correct, I was in my first year there. But, no, I have not returned to school. I took a leave of absence.

Q. Thank you, Mr. Dunning.

THE COURT: You may step down, Mr. Dunning.

MS. BENNETT: Your Honor, at this time the State rests.

**High Impact VIS**

The Prosecution called the additional witness:

MS. BENNETT [Prosecutor]: The State calls Matthew Dunning.

MATTHEW DUNNING, having been first duly sworn, testified as follows:

DIRECT EXAMINATION BY MS. BENNETT:

Q. Mr. Dunning, could you please tell the court what happened on September 18, 2019?

A. Yes. On September 18, 2019, Mr. Taylor entered the Quick Stop gas station where I was working as a cashier. Mr. Taylor pulled a gun on me and yelled at me to open the register and put the money on the counter. I did what he wanted, but my hands were shaking. I asked him to stay calm, but he kept yelling. Then, he told me to get back, and after I did, he shot me. I couldn’t believe it, as scared as I was, I thought everything would be okay, but it wasn’t.

Q. And what did Mr. Taylor do after that?

A. Umm, well, I heard him grab the cash and run out the door.

Q. You “heard” him. You didn’t see him? What were you doing at that point?

A. Well, I was lying on the floor. I really thought that I was going to die. It certainly felt like it.

Q. Mr. Dunning, could you describe to us the impact this crime has had on you?

A. Things have been difficult. I used to love the quiet, but I hate the quiet now because it was quiet before he came into the store. I have to leave the TV on to sleep and I still jump at every loud noise. I can still feel the pain and fear I had when I realized that I had been shot. I was diagnosed with post-traumatic stress disorder a few months after I was shot. I have been in counseling since, but I still have not been able to return to work. The days are a lot harder than they used to be.

Q. Have you been able to return to school? You attend Rutgers Medical School, is that correct?

A. Yes, that is correct, I was in my first year there. But, no, I have not returned to school. I had to take a leave of absence. I didn’t want to, but I didn’t really have any other option... People really aren’t joking when they say that keeping up in med school is like drinking from a fire hose. After all that time in the hospital and at home recovering, I’d missed too much to catch up… I won’t be graduating with my class. It seems like my career has been put on hold.

Q. Thank you, Mr. Dunning.

THE COURT: You may step down, Mr. Dunning.
MS. BENNETT: Your Honor, at this time the State rests.

**Appendix E**

**Sentence Unscrambling Task**

Before reading the trial, we would like you to complete the following cognitive task.


For each set of words below, make a grammatical sentence and write it down in the space provided. For each set of words, there is ONE word that is NOT needed in the sentence.


Example: Flew eagle the plane around --> The eagle flew around.

**Perspective Taking with Defendant Condition**

empathize I him take with

________________________________________________________________

understand perspective he the I defendant's

________________________________________________________________

John her Heather relate to can

________________________________________________________________

can to took relate defendant I the

________________________________________________________________

I defendant's picked considered the situation

________________________________________________________________

understand I mother defendant's mindset the

________________________________________________________________

from see the where defendant I him is coming

________________________________________________________________

my heard he saw side

________________________________________________________________

with view I the empathize defendant

________________________________________________________________

story the hear side him I defendant's of

________________________________________________________________

perspective I understand Jeremy's picture

________________________________________________________________

myself I in shoes the can pants imagine defendant's

________________________________________________________________

he Sally sympathizes Harry with

________________________________________________________________

defendant's context I see guess the in the situation

________________________________________________________________

himself I defendant sympathize with the

________________________________________________________________

**Neutral (No Perspective Taking) Condition (taken from Chartrand & Bargh, 1996)**

ball throw toss silently the

________________________________________________________________

somewhat prepared I was refer

________________________________________________________________

picked throw apples hardly the

________________________________________________________________

they obedient him often meet

________________________________________________________________

helpless it hides there over

________________________________________________________________

send I mail it over

________________________________________________________________

a smile what parrot great

________________________________________________________________

ball the hoop toss normally

________________________________________________________________

saw hammer the train he

________________________________________________________________

maintain she to composure try

________________________________________________________________

have wing a butterfly I

________________________________________________________________

sky the seamless red is

________________________________________________________________

a have June holiday wedding

________________________________________________________________

she line leads the children

________________________________________________________________

***** Chartrand, T. L. & Bargh, J. A. (1996). Automatic activation of impression formation

and memorization goals: nonconscious goal priming reproduces effects of explicit task instructions. *Journal of Personality and Social Psychology, 71*(3), 464-478. DOI: 10.1037/0022-3514.71.3.464
